# Hormonal pleiotropy helps maintain queen signal honesty in a highly eusocial wasp

**DOI:** 10.1038/s41598-017-01794-1

**Published:** 2017-05-10

**Authors:** Ricardo Caliari Oliveira, Ayrton Vollet-Neto, Cintia Akemi Oi, Jelle S. van Zweden, Fabio Nascimento, Colin Sullivan Brent, Tom Wenseleers

**Affiliations:** 1 Laboratory of Socioecology and Social Evolution, Zoological Institute, KU Leuven Leuven, Belgium; 2 Departamento de Biologia, Faculdade de Filosofia, Ciências e Letras de Ribeirão Preto, Universidade de São Paulo, Ribeirão Preto, SP Brazil; 3 Arid Land Agricultural Research Center, USDA-ARS, Maricopa, Arizona USA

## Abstract

In insect societies, both queens and workers produce chemicals that reliably signal caste membership and reproductive status. The mechanisms that help to maintain the honesty of such queen and fertility signals, however, remain poorly studied. Here we test if queen signal honesty could be based on the shared endocrine control of queen fertility and the production of specific signals. In support of this “hormonal pleiotropy” hypothesis, we find that in the common wasp, application of methoprene (a juveline hormone analogue) caused workers to acquire a queen-like cuticular hydrocarbon profile, resulting in the overproduction of known queen pheromones as well as some compounds typically linked to worker fertility. By contrast, administration of precocene-I (a JH inhibitor) had a tendency to have the opposite effect. Furthermore, a clear gonadotropic effect of JH in queens was suggested by the fact that circulating levels of JH were ca. 2 orders of magnitude higher in queens than those in workers and virgin, non-egg-laying queens, even if methoprene or precocene treatment did not affect the ovary development of workers. Overall, these results suggest that queen signal honesty in this system is maintained by queen fertility and queen signal production being under shared endocrine control.

## Introduction

One of the defining characteristics of eusociality is the spectacular reproductive division of labor between fertile queens and mostly nonreproductive workers^1^. In many species of social insects, it has been shown that the queen can help to maintain this reproductive division of labor through the emission of specific signals, which reliably indicate her greater fertility, and to whom the workers respond by remaining sterile^2, 3^. The best known example undoubtedly is the queen mandibular pheromone 9-ODA released by honeybee queens, which not only plays a role during mating, but also stop the workers from reproducing^4^. Following extensive research on the honeybee queen pheromone system, many recent studies have shifted their attention to specific queen-characteristic cuticular hydrocarbon compounds (CHCs), which, based on bioassays with synthetic compounds, have been shown to act as conserved queen- and fertility-signals across several independently evolved eusocial lineages, including ants^5, 6^, wasps^7^ and bumblebees^8, 9^. Comparative analyses have further shown that hydrocarbons are the most common class of compounds that appear as queen-characteristic signals in social Hymenoptera^8, 10^.

Recently, it has been suggested that the convergent evolution of hydrocarbon queen pheromones across several independently evolved lineages may be driven by the fact that specific CHCs tend to correlate with reproductive state in both social and non-social insect species^10–16^, and that this would predispose them to be co-opted as honest signals of fertility^10, 17–19^. The way in which this tight linkage between fertility and the production of specific hydrocarbons signals would come about is still under debate, but one popular theory has suggested that this could be explained by fertility and hydrocarbon signal production being under shared endocrine control^13^. In support of this hypothesis based on “hormonal pleiotropy”^20^, it has been shown that the production of fertility-linked cuticular compounds is under juvenile hormone (JH) in fruit flies^21^, cockroaches^22^ and burying beetles^23^ as well as in primitively eusocial Ponerine ants^17, 24^ and Polistine wasps^25–27^, and that juvenile hormone also plays a key role in the reproduction of several of these species by acting as a gonadotropin^27–31^. At present, it remains unknown, however, if also in highly eusocial species with strong queen-worker caste dimorphism, fertility and the production of queen signals remain under shared JH control, and if hormonal pleiotropy therefore contributes to the maintenance of queen signal honesty. With the available data, such a hypothesis would be speculative at best. Indeed, although JH appears to act as a gonadotropin in bumblebees^32, 33^, several Polistine wasps^25–27, 34^ and fire ant queens^28^, JH in highly eusocial species such as honeybees, stingless bees and some ant species appears to have lost its primary role in regulating reproduction, and has secondarily acquired other roles^35, 36^, e.g. underlying polyphenisms in some ants^37^, driving age-related division of labor among the workers in honeybees and some Polistine wasps^35, 38, 39^, or underlying caste determination in several bees, wasps and ants^40–49^. Furthermore, no experimental work has established that JH regulates the production of cuticular hydrocarbon queen signals in any highly eusocial species, such as the Vespine wasp species from which bioactive hydrocarbon queen signals have been isolated^7, 8, 50^.

The aim of the present study, therefore, was to test whether hormonal pleiotropy also underlies the maintenance of queen signal honesty in the highly eusocial common wasp *Vespula vulgaris*. This species has steadily emerged as a model system in the study of social insect hydrocarbon queen pheromones^13, 51^, as four queen-characteristic hydrocarbon compounds, *n*-C_27_, *n*-C_28_ and *n*-C_29_ and 3-MeC_29_, have unambiguously been shown to act as sterility-inducing queen signals in this species^8^, and that on top of that, 3-MeC_29_ has also been shown to act as a queen egg-marking pheromone that enables the workers to recognize queen-laid eggs from worker-laid eggs, thereby allowing them to selectively remove or “police”^52^ eggs laid by rare reproductive “cheater” workers^50^. To test the role of JH in regulating these and other putative queen-signals^53^, we test if we could induce their production by treating workers with the JH analog methoprene and obtain a reverse effect by treating them with the JH inhibitor precocene-I^54, 55^. Subsequently, we determine if JH indeed acts as a gonadotropin in queens or workers in this species by comparing the JH hemolymph titers of reproductive queens with that of non-reproductive virgin queens and non-reproductive workers, as well as by testing whether the methoprene or precocene-I treatments affected the workers’ ovary development.

## Results

### Juvenile hormone control of queen- and fertility-linked hydrocarbon signals

**Figure 1:**
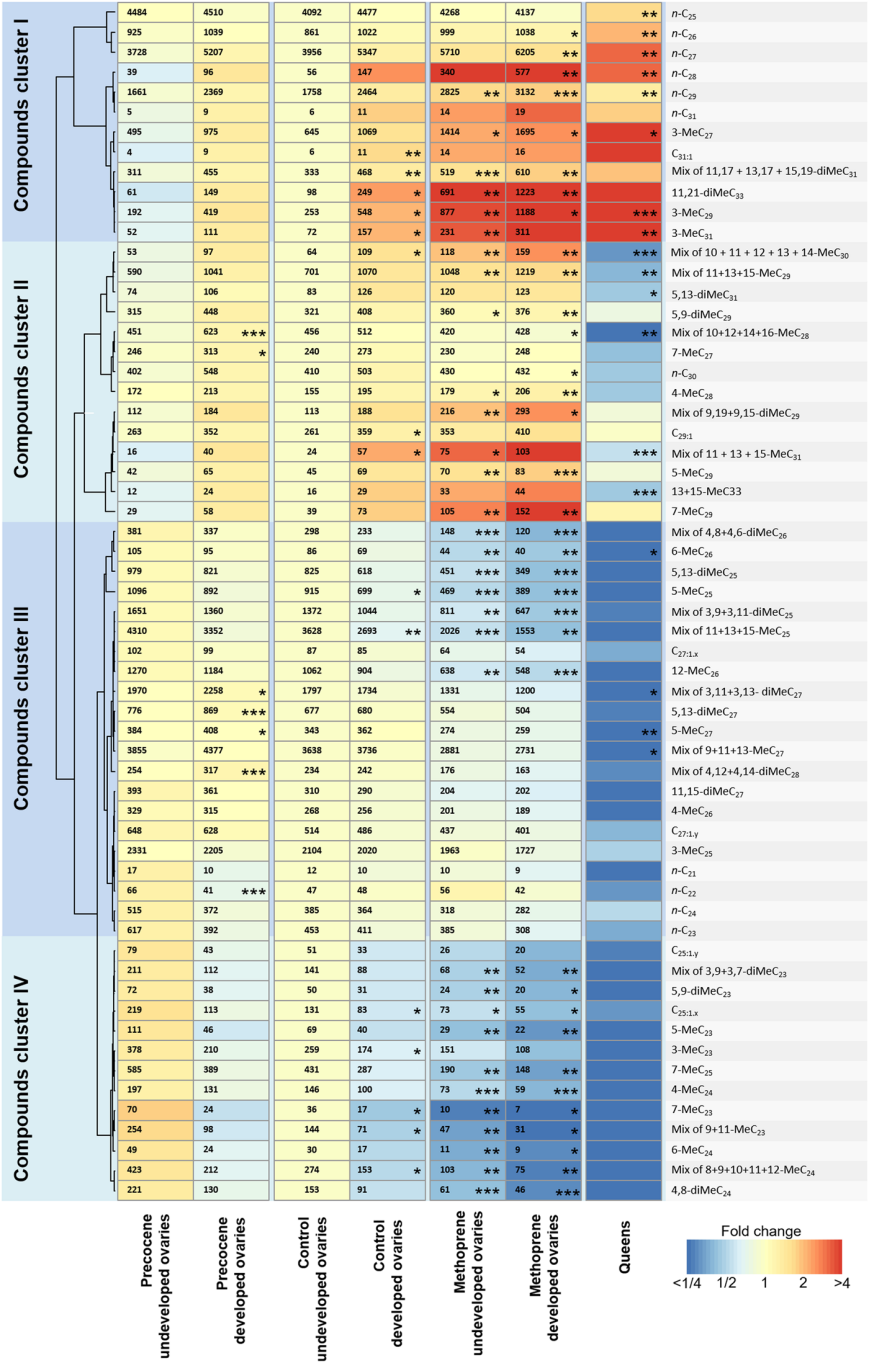
Heatmap showing JH-induced changes in cuticular hydrocarbon profiles. Effect of treating workers with the JH-analog methoprene and the JH inhibitor precocene-I on cuticular hydrocarbon profiles and known queen and fertility signals. Colors indicate the fold difference in relative abundance of each CHC peak compared to control workers with undeveloped ovaries. Hence, a value of two implies that the relative abundance of a given compound would be twice as high as in the reference acetone solvent control group. Columns of the heatmap correspond to the different treatments, which were split up by observed ovary development. The compound cluster analysis was based on a UPGMA hierarchical clustering using one minus the Pearson correlation as the distance metric. Values indicate mean absolute quantities per worker in ng and asterisks indicate significance levels for the following contrasts: control developed vs control undeveloped, precocene developed vs control developed, precocene undeveloped vs control undeveloped, methoprene developed vs control developed, methoprene undeveloped vs control undeveloped and queens vs methoprene developed, derived from a LMM with Benjamini & Yekutieli^82^ FDR *p* value correction: *p < 0.05, **p < 0.01, ***p < 0.001. For detailed statistical results see Supplemental Table S2.

**Figure 2:**
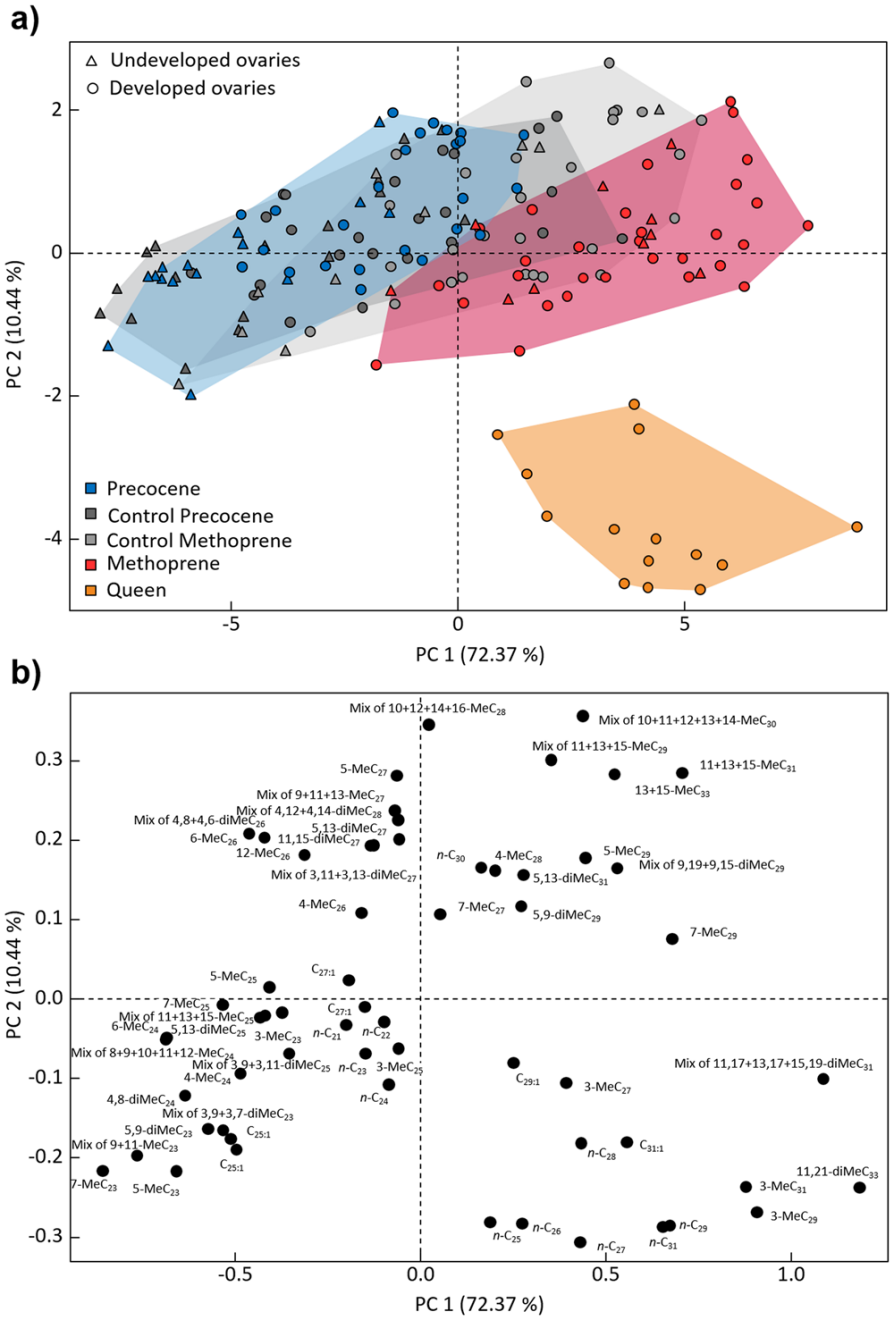
Principal component analysis of JH-induced changes in cuticular hydrocarbon profiles. (**a**) PC factor scores (calculated from a PCA on Aitchison transformed relative compound abundances) show that treatment with the JH-analog methoprene (red) induced worker profiles to become more queen-like (orange), whereas treatment with the JH inhibitor precocene-I had a slight reverse effect (*n* = 40 per group for workers and *n* = 14 for egg-laying queens). Workers with developed ovaries (circles) can also be observed to be more queen-like along PC axis 1 than workers with undeveloped ovaries (triangles). (**b**) Corresponding factor loadings for the different hydrocarbon compounds. The four known queen pheromones appear in the lower right quadrant (*n*-C_27_, *n*-C_28_, *n*-C_29_ and 3-MeC_29_).

**Figure 3:**
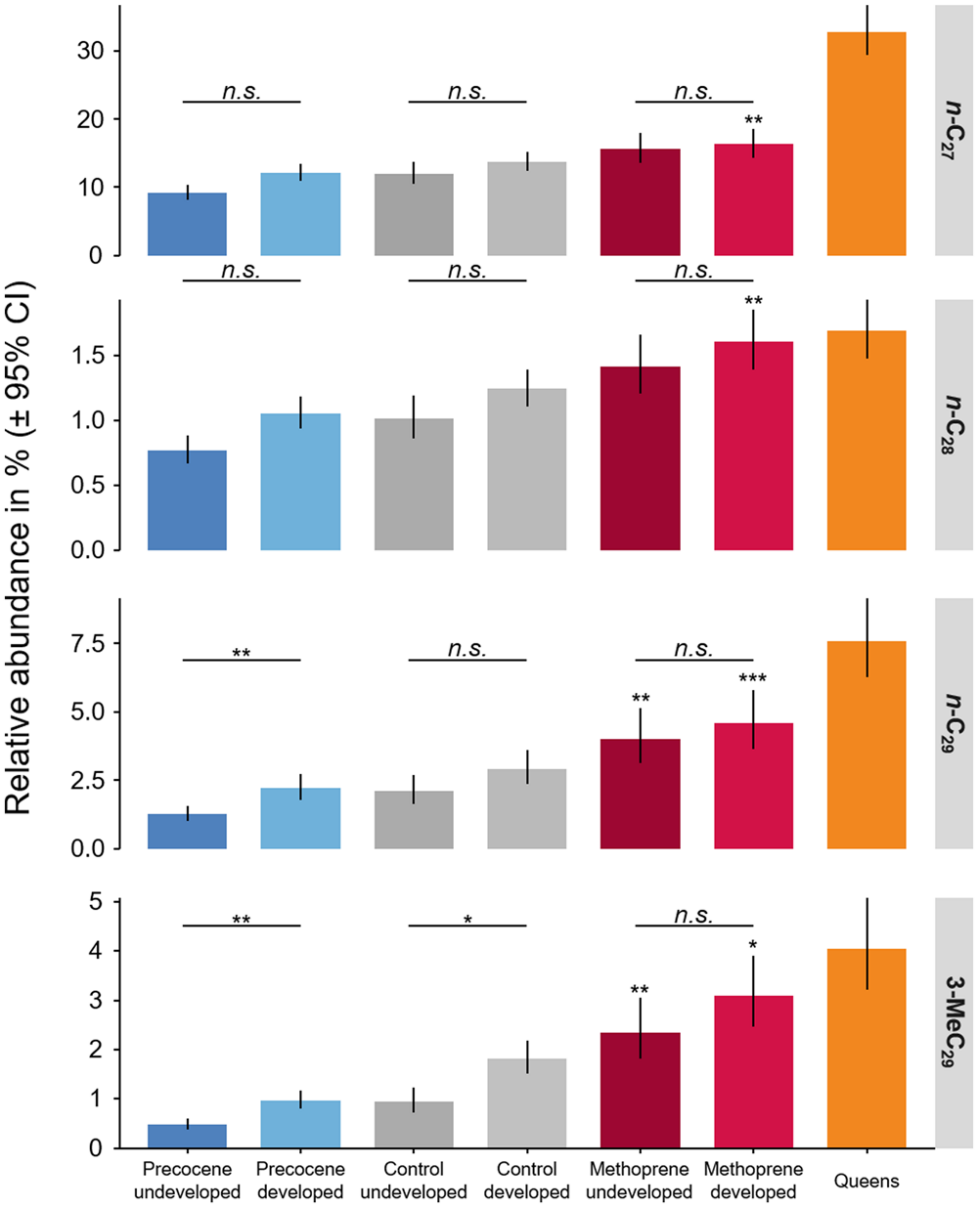
Juvenile hormone regulates known queen signals in the common wasp. Treatment of workers with the JH analog methoprene or the JH inhibitor precocene-I enhances or suppresses the production of four known sterility-inducing hydrocarbon queen pheromones (bars and whiskers = mean relative abundances and 95% confidence intervals calculated on log transformed relative abundance and then backtransformed to the original scale, asterisks above bars indicate the significance of contrasts with their respective control, whereas the asterisks above each pair of bars indicate differences in abundance between workers with or without developed ovaries of a given group; significance levels are based on linear mixed models on Aitchison transformed abundances, using Benjamini & Yekutieli^82^ FDR *p* value correction: *p < 0.05, **p < 0.01, ***p < 0.001).

To test if juvenile hormone (JH), which typically acts as a gonadotropin in wasps^26, 27, 29, 34, 38, 56^ (cf. results below), also controls the production of queen- or fertility-linked cuticular hydrocarbon signals, we performed experiments in which we treated common wasp workers that had been isolated from their mother queen with the JH analog methoprene and the JH inhibitor precocene-^51, 52^. In line with prediction, we find that 13 days post-treatment, workers treated with methoprene had acquired a CHC profile that had become queen-like and which vastly differed from the acetone solvent control group, whereas precocene-I tended to have the opposite effect (Figs 1–3). As expected under a queenless situation^57, 58^, some of the workers (42.5% across the different treatment groups) developed their ovaries over the duration of our experiments (cf. results below), and to allow for possible differences in the cuticular hydrocarbon profiles of both sets of workers these were considered separately in our analysis.

In the methoprene treated group, both workers with fully developed and undeveloped ovaries significantly upregulated more than 30 out of the 60 CHCs compared to the control (linear mixed models on Aitchison transformed relative abundances with FDR *p*-value correction, Fig. 1). Compounds that were significantly overproduced were mostly long-chain linear alkanes and 3-methyl-branched alkanes, as well as a few other monomethyl and dimethyl-branched compounds with chain lengths of 26 to 31 carbon atoms (Fig. 1). Significantly, these included all previously identified bioactive queen pheromones of this species^8^, and which are known to suppress worker reproduction, namely *n*-C_27_, *n*-C_28_, *n*-C_29_ and 3-MeC_29_ (cf. section below).

In the precocene treated group, by contrast, only seven compounds in workers with developed ovaries and none in workers with non-developed ovaries showed any significant changes (Fig. 1). Despite the relatively limited number of compounds that were strongly affected by the precocene treatment, the effect was clearly in the opposite direction as that of the methoprene treatment. This can be seen from the fact that the log_2_ fold difference in the change in relative abundance induced by the methoprene treatment compared to the control across all compounds was strongly negatively correlated with the change induced by the precocene treatment (for workers with undeveloped ovaries: Pearson *R* = −0.925, *t* = −18.54, *p* < 0.0001; for workers with developed ovaries: Pearson *R* = −0.924, *t* = −18.36, *p* < 0.0001). Hence, the two different treatments altered the profile of the workers in exactly opposite directions, implying that compounds that were upregulated in one group were downregulated in the other and vice versa.

Comparison of the control group workers with developed and undeveloped ovaries demonstrates that some compounds also displayed significant differences linked to the workers’ reproductive state (Figs 1–3). One of the known queen pheromones of this species, 3-MeC_29_, for example, was overproduced in workers with developed ovaries compared to those with undeveloped ones (Fig. 1). Indeed, also from the methoprene treatments, it is clear that JH regulated not only the production of caste-specific signals but also that of fertility signals linked to worker reproductive state. This is clear from the fact that methoprene treated workers appeared to acquire an overall CHC profile which was intermediate between that of reproductive workers and queens (Fig. 1). In fact, in the heatmap, a UPGMA cluster analysis on the compound abundances revealed a number of clear groups with compounds characteristic for either queens (Fig. 3, Compound cluster 1) or workers (Fig. 3, Compound cluster 4) (i.e. caste- and fertility-linked signals) as well as of compounds which were either overproduced or underproduced by reproductive workers (Fig. 3: Compound clusters 2 and 3) (i.e. worker fertility-linked signals). When the expression of individual compounds in queens were compared to methoprene treated workers with developed ovaries, only 18 CHCs significantly differenced in abundance with levels observed in queens, with some compounds being upregulated and some others downregulated (Fig. 1).

Multivariate analyses further support the generic patterns described above. A Permanova analysis confirms that there were highly significant differences in the multivariate CHC profiles across the different treatment groups (*F* = 37.76, *p* < 0.0001). In addition, a Principal component analysis on Aitchison transformed relative abundances revealed a first PC axis which explained 72% of the total variance that was strongly linked to both caste (queen-worker differences) and worker fertility (in the control and precocene-I treatment groups), and a second PC axis that explained 10% of the total variance and which was negatively correlated with the production of caste-specific queen signals (Fig. 2a). The hormone and hormone inhibitor treatments mainly caused a shift along the first PC axis. As expected then, the known bioactive queen pheromones^8^ *n*-C_27_, *n*-C_28_, *n*-C_29_ and 3-MeC_29_, together with some other linear alkanes and 3-methylalkanes, all appear in the lower right quadrant of the factor loadings diagram (Fig. 2b). Notably, the PCA analysis confirms the general pattern that the methoprene treated workers acquired a CHC profile that was intermediate between that of queens and workers, whereas precocene-I induced a slight shift in the opposite direction, resulting in the exaggeration of compounds characteristic for sterile workers (Fig. 2a). A discriminant analysis on principal components (DA-PC) based on the four first principal components enabled the correct assignment of all queens and methoprene treated workers, whereas for the precocene-I treated group 35 out of 40 samples (87.5%) could be correctly classified.

### Juvenile hormone control of known queen pheromones

When the relative expression levels of the four known bioactive common wasp queen pheronomes (*n*-C_27_, *n*-C_28_, *n*-C_29_ and 3-MeC_29_) are graphed separately across the different treatment groups, we find that, as predicted, all four and two out of four of the compounds (*n*-C_29_ and 3-MeC_29_) are significantly upregulated in the methoprene-treated worker groups with developed and undeveloped ovaries, respectively (Fig. 3, LMMs on Aitchison transformed abundances with FDR *p*-value correction across all compounds, methoprene-treated workers with developed ovaries: *n*-C_27_: *z* = 3.828, *p* = 0.006, *n*-C_28_: *z* = 3.841, *p* = 0.0002, *n*-C_29_: *z* = 4.675, *p* = 0.001, 3-MeC_29_: *z* = 3.927, *p* = 0.018; methoprene-treated workers with undeveloped ovaries: *n*-C_29_: *z* = 4.134, *p* = 0.003, 3-MeC_29_: *z* = 4.248, *p* = 0.002, for detailed statistical results see Supplemental Table S3). Hence, methoprene treatment of workers resulted in them acquiring more queen-like expression levels for the known queen pheromones^8^. By contrast, precocene-I treatment had the reverse effect, even if this effect was not significant after correcting *p* values for multiple testing. Together, these data clearly show that juvenile hormone regulates the production of known queen signals in the common wasp.

On top of these treatment-induced shifts towards or away from queen-like profiles, workers with developed ovaries also produced significantly higher amounts of *n*-C_29_ and of 3-MeC_29_ in the precocene-I treated group (LMMs, *z* = 4.484, *p* = 0.002 and *z* = 4.579, *p* = 0.002, respectively) and of 3-MeC_29_ in the control group (LMMs, *z* = 3.542, *p* = 0.039, Fig. 3). Nevertheless, the relative amounts produced by workers with developed ovaries was still much lower than that observed in queens (Fig. 3).

### Gonadotropic effect of juvenile hormone in queens but not workers

In a last set of experiments, we determined if the clear effect that JH analogs or JH inhibitors had on the production of queen and fertility-linked signals were matched by JH actually having a gonadotropic effect in either queens or workers. To determine the gonadotropic effect of JH in queens, we determined the JH III hemolymph titers in mature egg-laying common wasp queens, non-reproductive virgin queens and workers, whereas the gonadotropic effect of JH in workers was inferred from dissecting a randomly sampled subset of the workers derived from the bioassay described above (*n* = 40 per treatment group).

**Figure 4:**
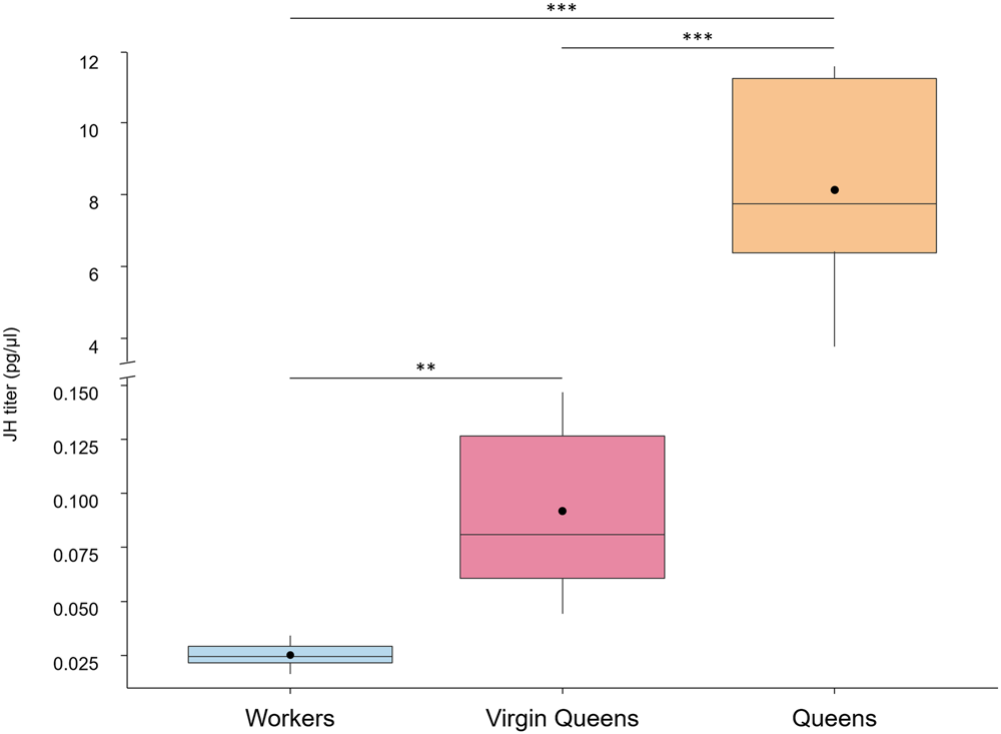
Juvenile hormone titers support a gonadotropic effect in common wasp queens. Circulating JH levels in pg/μl are 88 times higher in mature, egg-laying queens than in non-reproductive virgin queens, and 325 times higher than in nonreproductive workers (black circles and whiskers = averages and 95% confidence intervals calculated on a log scale and then backtransformed to the original scale, boxes = quartiles, horizontal bars = medians, *n* = 5 per group, but using pools of 5 individuals per sample for the workers; significance levels based on Anova and Tukey posthoc tests performed on log transformed data: **p < 0.01, ***p < 0.001).

Analysis of the JH III hemolymph titers demonstrates that the JH titers were strongly correlated with reproductive status, thereby supporting that JH is a gonadotropic hormone in the queens of this species. In fact, egg-laying queens had an average of 8.13 pg/μl of circulating JH, which was 88 times greater than the amount observed in non-reproductive virgin queens (0.092 pg/μl, Anova on log transformed JH titers with Tukey post-hoc correction, *p* < 0.0001) and 325 times greater than the amount observed in (nonreproductive) workers (0.025 pg/μl, *p* < 0.0001) (Fig. 4). Although the concentration of JH in the hemolymph of virgin queens was much lower than in egg-laying queens, it was still significantly higher than that of workers (Anova on log transformed JH titers with Tukey post-hoc correction, *p* = 0.0017).

**Figure 5:**
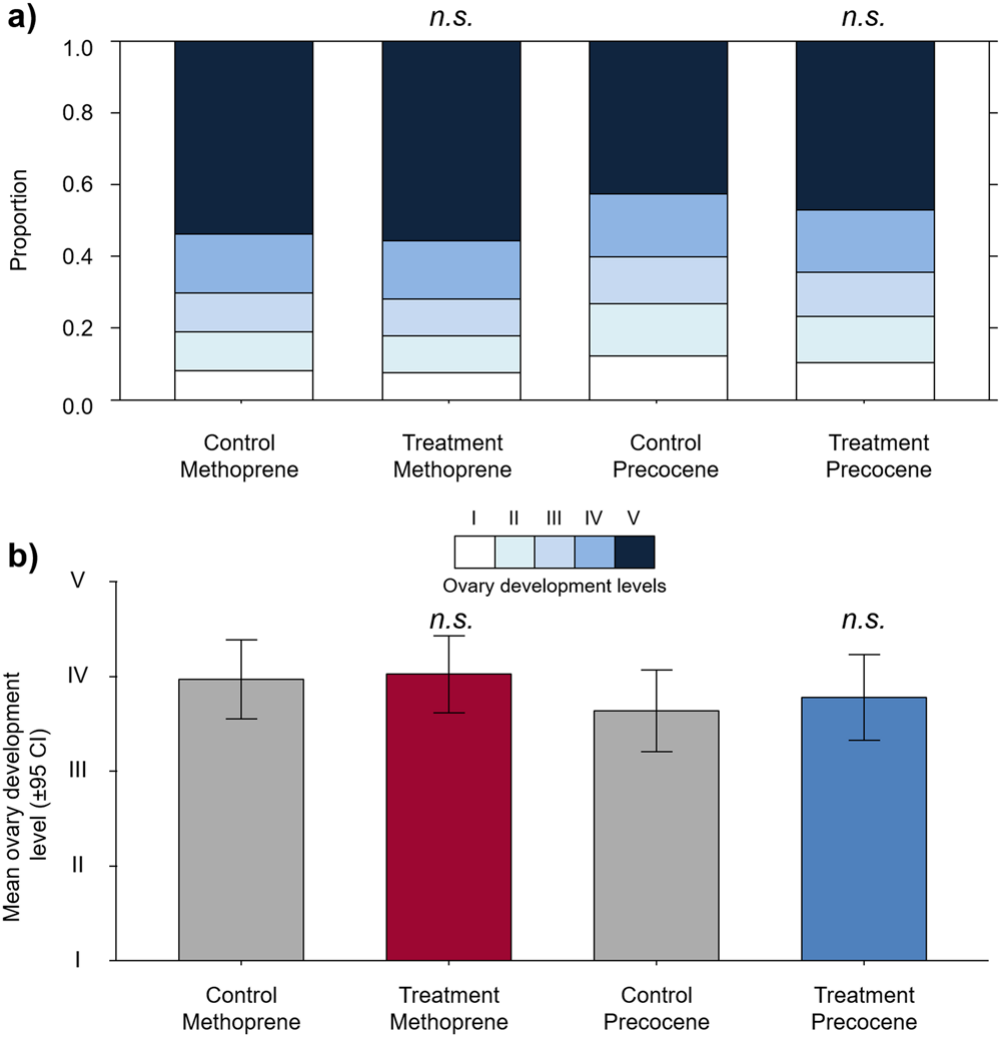
Juvenile hormone does not have a gonadotropic effect in workers. Treatment of workers with the JH analog methoprene or the JH inhibitor precocene-I did not affect rates of worker ovary development compared to their respective control when scored on a five point scale (I = undeveloped, V = fully developed) after a period of 13 days (panel a = stacked bar charts, panel b = mean ovary development and 95% confidence intervals shown on an ordinal scale, n.s. = non-significant differences based on an ordered logistic model, *n* = 40 workers per treatment group).

In contrast to the clear evidence for a gonadotropic effect of JH in queens, dissection of workers from the bioassay experiment revealed that topical application of neither methoprene or precocene-I had any effect on levels of worker ovary development (Fig. 5), despite the fact that a high proportion of workers (42.5%) developed their ovaries across all treatment groups during the experimental period (Fig. 5). This was shown from the fact that when the workers’ ovary development was scored on a 5-point scale (see methods), no quantitative differences in worker ovary development were observed either among the methoprene or precocene-treated groups and their respective controls (ordinal logistic model with Tukey posthoc correction, methoprene: *z* ratio = −0.181, *p* = 0.998; precocene: *z* ratio = −0.447, *p* = 0.970) or between the two treatments (*z* ratio = 0.785, *p* = 0.861). From this, we can conclude that although JH likely had a gonadotropic effect in common wasp queens, it did not have this effect in workers.

## Discussion

Overall, this study provides clear evidence that both queen fertility and the production of queen signals are under shared juvenile hormone (JH) control in the highly eusocial Vespine wasp *Vespula vulgaris*. Hence, hormonal pleiotropy likely contributes to keep queen signals honest and reliable in this species. The most striking observation in our experiments was that administering the JH analog methoprene or the anti-JH precocene readily caused the worker profiles to become more or less queen-like, and that the methoprene treatment caused workers to greatly overproduce all four previously reported hydrocarbon queen signals^8, 50^, *n*-C_27_, *n*-C_28_, *n*-C_29_ and 3-MeC_29_, which in this species are characteristic for queens and are known to serve a function in suppressing worker reproduction^8^ or acting as a queen-egg marking pheromone^50^, thereby facilitating the policing of worker-laid eggs^59^. Furthermore, a gonadotropic effect of JH was inferred for queens based on the fact that circulating JH titers were 88 times greater in egg-laying common wasp queens than in non-reproductive virgin queens, and 325 times greater than in non-reproductive workers. By contrast, no gonadotropic effect of JH was apparent in workers, since neither the methoprene nor the precocene-I treatment had any effect on observed levels of worker ovary development compared to the control. These results are consistent with other data from primitively eusocial Polistine wasps, where several studies have found evidence for JH regulating the reproduction of queen foundresses but not that of the offspring workers, and with JH in workers instead appearing to regulate the age-related division of labor in these species (In *Polistes dominula* ^29, 39^ and *P. canadensis* ^38^). In addition, a gonadotropic effect of JH has also been reported in bumblebees^33^ and fire ant queens^28^, though not in honeybees, where JH instead is involved in caste determination^60^ and in regulating age-related division of labor in workers^35, 61^, and where JH titers in egg-laying queens are very low^62^. At present, the role of JH in common wasp workers would require further study. Nevertheless, if it would turn out to be the case that JH regulated age-related division of labor also in *V. vulgaris*, then this would in fact imply that workers would not be able to readily mimic queen signals, e.g. as a way for reproductive workers to make their eggs smell like those of the queen and avoid their eggs being policed^63, 64^. Workers of *V. vulgaris* police the eggs of reproductive workers, which use their chemical signature to differentiate between queen and worker laid eggs in such an effective way that in queenright conditions almost none of workers’ eggs thrive^59, 65–67^. Based on our results, the upregulation of JH in egg-laying workers that would be required to mimic the queen’s signal would then also affect division of labor, and result in the progression to a forager role. In addition, our results suggest that egg-laying workers would be unlikely to succeed in fully mimicking the queen’s signals, since methoprene treatment of workers still resulted in a profile that was intermediate between that of normal workers and queens, and several compounds were also found to be unique to reproductive workers (compound cluster II in Fig. 1).

The evidence that we found for both queen signals and queen fertility to be under shared JH control is interesting given that an increase in JH titers has earlier also been shown to correlate with changes in the CHC profiles in several different polistine wasp species, including *Polistes dominula*, *P. smithii*, *Synoeca surinama* and *Polybia micans* and that JH – with the possible exception of *P. smithii* –also influenced ovary development in dominant egg-layers in all these species^25–27, 68^ (for further evidence of a gonadotropic effect of JH in Polistine wasp foundresses see refs 34, 38). In addition, as mentioned before, JH-induced changes in cuticular profiles in combination with JH-induced gonadotropic effects have also been documented in several solitary insect species^16, 21, 30, 31, 69–72^. This implies that hormonal pleiotropy may well lie at the basis of the observed honesty and reliability of cuticular hydrocarbon fertility signals across several major insect groups, and that this may have predisposed such cues to have repeatedly been adopted as queen signals^8, 13^.

In addition to the observed conservation of shared JH control of queen signals and queen fertility in wasps, it is interesting to note that the main compounds which were regulated by JH, including *n*-C_29_, 3-MeC_29_ and *n*-C_31_, have earlier been identified as compounds that showed a strong conservation as queen- and fertility-signals across different species of Vespine wasps^51^. These results suggest the presence of a conserved underlying genetic pathway by which JH acts on hydrocarbon biosynthesis. Given that methoprene-treated workers mainly overproduced longer-chain hydrocarbons (backbone chain lengths of 26 to 31 carbon atoms) and underproduced shorter-chain compounds (backbone chain lengths of 23 to 26 carbon atoms), an effect of JH on the chain lengthening of the fatty acid CHC precursors via a microsomal fatty acyl-CoA elongase would seem likely^11, 73, 74^. Nevertheless, detailed characterization of the pathways by which JH acts on the production of queen and fertility signals awaits further study.

Overall, we think that our results on the shared juvenile hormone control of queen fertility and queen signaling provides unique new insights in the way by which signal honesty in this system may be maintained and the factors that may have promoted ancestral fertility signals to be co-opted as queen signals. Future work could be targeted at gaining a better molecular understanding of the way in which JH jointly regulates fertility and signal production in the queen caste, as well as document the effects of JH in the worker caste in a larger set of highly eusocial insect species, in order to test the extent of any other possible pleiotropic physiological or behavioural effects.

## Methods

### Bioassays with JH analog methoprene and JH inhibitor precocene-I

**Figure 6:**
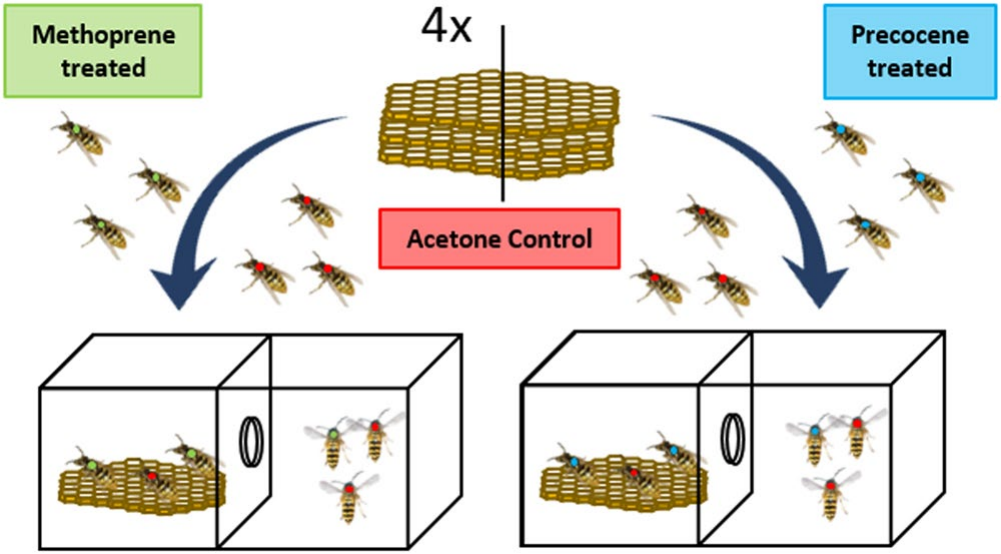
Scheme of the experimental setup. The combs of each of four natural colonies were divided into equal halves and put into separate boxes together with ca. 50 untreated workers. The abdomen of ca. 80 newly emerged workers per box and treatment was then topically treated once with 5 µl of either 20 µg/µl methoprene in acetone solution (green), 6 µg/µl precocene-I in acetone solution (blue), or an acetone solvent control (red). After two weeks the workers were frozen at −20 °C for later analysis of the CHCs.

In a first experiment, we tested if topical application of the juvenile hormone (JH) analog methoprene induced *Vespula vulgaris* common wasp workers to acquire a queen-like cuticular hydrocarbon profile, and if treatment with the JH inhibitor precocene-I had the reverse effect. In addition, we also tested for possible effects on worker ovary development, thereby enabling us to decide if JH acts as a gonadotropin in workers in this species. To this end, four *Vespula vulgaris* colonies were collected in the surroundings of Leuven, Belgium, in July and August 2014. In the laboratory, each colony was then anaesthetized with carbon dioxide and divided into two equal queenless halves, each containing ca. 50 adult workers and a single brood comb with eggs and larvae. Subsequently, each group served as a host group for either methoprene and acetone sham-treated control workers or precocene-I and control workers (Fig. 6). The combs were chosen to contain as few pupae as possible, to avoid new, untreated workers from emerging during the experiment later on. Colonies were kept in experimental wooden nest boxes (35 × 14.5 × 30 cm), which had two compartments: one where the nest comb was kept and another that allowed workers to be fed and forage for food. In the latter compartment, we provided sugar paste, water *ad libitum*, and six mealworms per day. The boxes were kept inside the laboratory at an average temperature of 28 °C. The remaining combs containing pupae were kept in a wooden box to allow the emergence of the adults, which were subjected to different hormone treatments on the day they emerged. These newly emerged individuals were anaesthetized with carbon dioxide and divided into four equal groups per day. Workers were color marked with a different color per treatment group and treated via topical application on their abdomens with 5 µl of the following treatment solutions: 20 µg/µl solution of methoprene (SIGMA-ALDRICH, analytical standard) in acetone, 6 µg/µl solution of precocene-I (SIGMA-ALDRICH, analytical standard) in acetone, and two acetone solvent control groups. The chosen doses were based on typical levels used in comparable experiments in other related species^29, 56, 75^, with the precocene concentration used being slightly lower due to its greater toxicity documented in honeybees and other insects^76^. Prior to settling on the final dose used we confirmed that neither methoprene nor precocene-I had any toxic effects across three doses tested, which included the dose that was actually used (see toxicity tests below). In each experimental box, one of the treatments was always introduced together with a matching acetone control group (Fig. 6). This process was repeated over two consecutive days, resulting in ca. 60 individuals being introduced per treatment group across all eight replicate boxes deriving from the four study colonies. After 13 days, which exceeds the time of 10 days required for workers to active their ovaries and commence egg-laying in this species^57^, all individuals were freeze killed at −20 °C. Subsequently, we randomly selected five individuals per treatment per box (N = 40 workers per treatment) for further cuticular hydrocarbon (CHC) analysis and dissections to assess the development of their ovaries and test whether or not JH acts as a gonadotropic hormone in workers (see below).

### Toxicity tests

Prior to the beginning of the bioassay, we performed a pilot experiment to make sure that neither methoprene or precocene-I had any toxicity effects around the doses that we planned on using. To this end, we set up a colony with ca. 80 newly emerged workers (placed under identical conditions as described above), of which 10 each were treated once with a dose of 5 µl of 5 µg/µl, 10 µg/µl or 20 µg/µl of methoprene, ca. 10 each were treated with a dose of 5 µl of 2 µg/µl, 6 µg/µl or 15 µg/µl of precocene-I, 10 were treated with acetone solvent and 10 were left untreated. Subsequently, the survival of these workers was measured at 3 and 6 days following treatment. Across all treatments, survival was high, with 65 out of 77 workers (84%) surviving at day 3 and 57 out of 77 workers (75%) surviving at day 6. A Cox proportional hazards regression model, fitted using the *coxph* function in the *survival* package within R version 3.2.4^77^, followed by pairwise Tukey posthoc tests, performed using the *lsmeans* package, revealed that there were no significant differences in survival among any of the treatment groups (raw data and complete statistics results are given in Tables S4 and S5 in supplemental material). Hence, we could safely conclude that there was no toxicity of either methoprene or precocene-I at the doses that we used.

### Chemical analysis of CHCs

To determine the cuticular hydrocarbon (CHC) profiles of the workers from the bioassay above and assess how queen-like they were, we extracted 40 workers per treatment group as well as 14 mother queens (obtained from nests collected the same year) in HPLC-grade pentane for 10 mins and analyzed their profiles using GC/MS analysis. The total extraction volume was 800 µl for workers and 2 ml for queens. Following extraction, samples were evaporated at room temperature under a flow cupboard and resuspended in 100 µl pentane for workers and 1 ml pentane for queen samples. Samples were analyzed on a gas chromatograph (Thermo Fisher Scientific Trace 1300 series) coupled with a mass spectrometer (Thermo Fisher Scientific ISQ series MS). We used an injection volume of 1 µL and splitless injection with an inlet temperature of 320 °C. The initial temperature of 70 °C was held for 2 min, then increased to 200 °C at a rate of 20 °C min^−1^, to 250 °C at a rate of 3 °C min^−1^, and finally to 320 °C at a rate of 5 °C min^−1^ which was held for 3 minutes. Helium was used as a carrier gas at a constant flow rate of 0.9 mL min^−1^. The electron ionization voltage was auto-tuned to enhance the acquisition performance according to the molecular weight of the compounds and the ion source temperature and MS transfer line temperature were both set to 300 °C. The column used was a Restek RXi-5sil MS 20 m with an internal diameter of 0.18 mm and a film thickness of 0.18 µm. Peaks in the total ion chromatogram were aligned and integrated using a custom R script (available on request). External linear alkane ladders containing *n*-heptane (C_7_) up to *n*-tetracontane (C_24_) injected at three different concentrations (0.1 µg/µl, 0.01 µg/µl and 0.001 µg/µl) were used to perform absolute quantification of all hydrocarbons. For each compound, we used the nearest eluting linear alkane to construct a calibration curve, using a linear regression on a log(concentration) vs. log(peak area) scale (in all cases there was excellent linearity on a log-log scale). For all peaks we calculated spline-interpolated retention indices^78^, which together with the expected hydrocarbon fragmentation patterns enabled all cuticular hydrocarbon compounds to be unambiguously identified.

We used linear mixed models on Aitchison^79^ (centered log-ratio) transformed absolute abundances to assess the influence of each of the treatments on the relative amounts of each CHC that was produced, as is standard in compositional data analysis^79^. In this analysis, treatment in interaction with ovary development status (fully developed or not) was coded as a fixed factor (resulting in treatment levels control developed, control undeveloped, methoprene developed, methoprene undeveloped, precocene developed, precocene undeveloped and queens), whereas colony ID was coded as a random intercept. Tests for differences in relative amounts were deemed more appropriate than tests for differences in absolute amounts given that all hydrocarbons present in common wasp workers were non-volatile and that social insects are known to perceive compound abundances relative to the rest of the compounds that are present^80^. In addition, large size differences among workers as well as among queens and workers would have confounded differences in absolute abundance. Raw absolute abundances, however, are given for future reference in supplementary Table S1. Pairwise posthoc tests were performed using the *glht* function in package *multcomp* ^81^ to test for the significance of the different treatments in relation to their respective control as well as between acetone control workers with or without developed ovaries and between queens and workers treated with methoprene. In these tests, *p*-values were corrected for multiple testing using Benjamini and Yekutieli^82^. A heatmap of the log_2_ fold differences in the relative abundance of each compound in each of the different groups in comparison to the acetone control workers with undeveloped ovaries was produced using the *pheatmap* package^83^. In this heatmap, compounds were clustered using UPGMA clustering and one minus the Pearson correlation as the distance metric. Finally, package *FactoMineR* ^84^ was used to perform an exploratory principal component analysis on Aitchison transformed abundances, and a discriminant analysis of principal components (DA-PC) with leave-one-out cross-validation was performed using package *MASS* ^85^.

### Gonadotropic effect of JH in workers

To test if our hormone treatments affected worker ovary development, and determine if JH had a gonadotropic effect on workers we dissected 40 randomly selected workers per treatment. Ovary development levels were quantified in a scale adapted from Duchateau and Velthuis^86^, in which stage I is when oocytes and trophocytes cannot be distinguished or oocytes and trophocytes can be differentiated but the oocyte is still small and spherical. In stage II the oocyte is no longer spherical but the trophocyte is longer than the oocyte. In stage III both the oocyte and trophocyte are enlarged and the oocyte has an oval shape. In stage IV the oocyte is clearly larger than the trophocyte which is degenerating. Finally, in stage V where a fully mature terminal oocyte can be seen with visible chorion and the terminal trophocyte is almost or entirely degenerated. To test whether ovary development levels were different across treatments, we ran an ordinal logistic model with function *polr* (package MASS ver. 7.3-45^85^) with the five different levels of ovary development as response factors and treatment (methoprene or precocene treated or control) specified as a fixed factor.

### Quantification of JH titers and gonadotropic effect of JH in queens

To test if JH had a gonadotropic effect in common wasp queens we compared circulating JH titers in mature, egg-laying queens with those in virgin, non-egg-laying queens and workers. Hemolymph JH content was determined using GC-MS, following previously established methods^87^. For each group, five samples were collected by piercing the cuticle and siphoning expelled hemolymph into a graduated glass capillary tube which was then blown into an individual glass vial containing 500 µl of acetonitrile (HPLC grade). Each sample contained 5 µL of hemolymph, which was collected readily from single queens but which required pooling the hemolymph fluid of 5 workers. Samples were kept at −80 °C until further processing. Prior to purification, farnesol (Sigma-Aldrich, St Louis, MO, USA) was added to each sample to serve as an internal standard. Samples were dried down by vacuum centrifugation, then extracted three times with hexane (HPLC grade). Each wash was added to borosilicate glass columns filled with aluminum oxide. In order to filter out contaminants, samples were eluted through the columns successively with hexane, 10% ethyl ether–hexane and 30% ethyl ether–hexane. After drying, samples were derivatized by heating at 60 °C for 20 min in a solution of methyl-d alcohol (Sigma-Aldrich) and trifluoroacetic acid (Sigma-Aldrich, St Louis, MO, USA). Samples were dried down, resuspended in hexane, and again eluted through aluminum oxide columns. Non-derivatized components were removed with 30% ethyl ether. The JH derivative was collected into new vials by addition of 50% ethylacetate–hexane. After drying, the sample was resuspended in hexane. Samples were then analyzed using an HP 7890 A Series GC (Agilent Technologies, Santa Clara, CA, USA) equipped with a 30 m × 0.25 mm Zebron ZB-WAX column (Phenomenex, Torrence, CA, USA) coupled to an HP 5975 C inert mass selective detector. Helium was used as a carrier gas. JH form was confirmed by first running test samples in SCAN mode for known signatures of JH 0, JH I, JH II, JH III and JH III ethyl; JH III was confirmed as the primary endogenous form in this species. Subsequent samples were analyzed using the MS SIM mode, monitoring at m/z 76 and 225 to ensure specificity for the d3-methoxyhydrin derivative of JH III. Total abundance was quantified against a standard curve of derivatized JH III, and adjusted for the starting volume of TF. Differences on the circulating levels of JH were assessed with an Anova on the log transformed concentrations of JH that was present (in pg/µl of hemolymph), followed by Tukey HSD posthoc comparison to test for pairwise differences among the different castes of differing reproductive status (egg-laying queens, virgin non-egg-laying queens and workers).

## Electronic supplementary material

Supplemental material

## References

[1] Wilson, E. O. *The Insect Societies*. (Belknap Press, 1971).

[2] Peeters, C. & Liebig, J. In *Organization of insect societies: from genome to socio-complexity* (eds Gadau, J. & Fewell, J.) 220–242 (Harvard University Press, 2009).

[3] Le Conte Y, Hefetz A (2008). Primer pheromones in social Hymenoptera. Annu Rev Entomol.

[4] Slessor KN, Winston ML, Le Conte Y (2005). Pheromone communication in the honeybee (*Apis mellifera* L.). J Chem Ecol.

[5] Holman L, Jorgensen CG, Nielsen J, d’Ettorre P (2010). Identification of an ant queen pheromone regulating worker sterility. Proc. R. Soc. London, Ser. B.

[6] Holman L, Lanfear R, d’Ettorre P (2013). The evolution of queen pheromones in the ant genus *Lasius*. J Evol Biol.

[7] Oi, C. A., Millar, J. G., van Zweden, J. S. & Wenseleers, T. Conservation of queen pheromones across two species of vespine wasps. *J Chem Ecol*, 1–6 (2016). 10.1007/s10886-016-0777-9 27722875

[8] Van Oystaeyen A (2014). Conserved class of queen pheromones stops social insect workers from reproducing. Science.

[9] Holman L (2014). Bumblebee size polymorphism and worker response to queen pheromone. PeerJ.

[10] Oliveira RC (2015). The origin and evolution of queen and fertility signals in Corbiculate bees. BMC Evol Biol.

[11] Blomquist, G. J. & Bagnères, A.-G. *Insect Hydrocarbons Biology, Biochemistry, and Chemical Ecology*. (Cambridge University Press, 2010).

[12] Liebig, J. In *Insect Hydrocarbons: Biology Biochemistry and Chemical Ecology* 254–281 (Cambridge University Press, 2010).

[13] Oi CA (2015). The origin and evolution of social insect queen pheromones: novel hypotheses and outstanding problems. BioEssays.

[14] Dillwith JW, Adams T, Blomquist GJ (1983). Correlation of housefly sex pheromone production with ovarian development. J Insect Physiol.

[15] Wicker C, Allon J-MJ (1995). Influence of ovary and ecdysteroids on pheromone biosynthesis in *Drosophila melanogaster* (Diptera: Drosophilidae). Eur. J. Entomol.

[16] Steiger S, Peschke K, Francke W, Müller JK (2007). The smell of parents: breeding status influences cuticular hydrocarbon pattern in the burying beetle *Nicrophorus vespilloides*. Proceedings of the Royal Society of London B: Biological Sciences.

[17] Holman L (2012). Costs and constraints conspire to produce honest signaling: insights from an ant queen pheromone. Evolution.

[18] Monnin T (2006). Chemical recognition of reproductive status in social insects. Ann Zool Fenn.

[19] van Zweden JS (2010). The evolution of honest queen pheromones in insect societies. Comm Int Biol.

[20] Flatt T, Tu MP, Tatar M (2005). Hormonal pleiotropy and the juvenile hormone regulation of *Drosophila* development and life history. BioEssays.

[21] Bilen J, Atallah J, Azanchi R, Levine JD, Riddiford LM (2013). Regulation of onset of female mating and sex pheromone production by juvenile hormone in *Drosophila melanogaster*. Proc Natl Acad Sci USA.

[22] Gu X, Quilici D, Juarez P, Blomquist G, Schal C (1995). Biosynthesis of hydrocarbons and contact sex pheromone and their transport by lipophorin in females of the German cockroach (*Blattella germanica*). J Insect Physiol.

[23] Haberer W, Steiger S, Müller JK (2010). (E)-methylgeranate, a chemical signal of juvenile hormone titre and its role in the partner recognition system of burying beetles. Anim Behav.

[24] Cuvillier-Hot V, Cobb M, Malosse C, Peeters C (2001). Sex, age and ovarian activity affect cuticular hydrocarbons in *Diacamma ceylonense*, a queenless ant. J Insect Physiol.

[25] Kelstrup HC, Hartfelder K, Wossler TC (2015). *Polistes smithii* vs. *Polistes dominula*: the contrasting endocrinology and epicuticular signaling of sympatric paper wasps in the field. Behav Ecol Sociobiol.

[26] Kelstrup HC, Hartfelder K, Nascimento FS, Riddiford LM (2014). Reproductive status, endocrine physiology and chemical signaling in the Neotropical, swarm-founding eusocial wasp *Polybia micans*. J Exp Biol.

[27] Kelstrup HC, Hartfelder K, Nascimento FS, Riddiford LM (2014). The role of juvenile hormone in dominance behavior, reproduction and cuticular pheromone signaling in the caste-flexible epiponine wasp, *Synoeca surinama*. Front Zool.

[28] Vargo EL, Laurel M (1994). Studies on the mode of action of a queen primer pheromone of the fire ant *Solenopsis invicta*. J Insect Physiol.

[29] Tibbetts EA, Izzo AS (2009). Endocrine mediated phenotypic plasticity: Condition-dependent effects of juvenile hormone on dominance and fertility of wasp queens. Horm Behav.

[30] Kelly TJ (1987). Juvenile hormone and ovarian maturation in the Diptera: A review of recent results. Insect Biochem.

[31] Martin D, Piulachs M-D, Belles X (1995). Patterns of haemolymph vitellogenin and ovarian vitellin in the German cockroach, and the role of juvenile hormone. Physiol Entomol.

[32] Bloch G (2000). Juvenile hormone titers, juvenile hormone biosynthesis, ovarian development and social environment in *Bombus terrestris*. J Insect Physiol.

[33] Shpigler H (2014). Gonadotropic and physiological functions of juvenile hormone in Bumblebee (*Bombus terrest*ris) workers. PLoS One.

[34] Tibbetts EA, Sheehan MJ (2012). The effect of juvenile hormone on *Polistes* wasp fertility varies with cooperative behavior. Horm Behav.

[35] Hartfelder K (2000). Insect juvenile hormone: from “status quo” to high society. Braz J Med Biol Res.

[36] Robinson GE, Vargo EL (1997). Juvenile hormone in adult eusocial Hymenoptera: Gonadotropin and behavioral pacemaker. Arch Insect Biochem Physiol.

[37] Lengyel F, Westerlund SA, Kaib M (2007). Juvenile hormone III influences task-specific cuticular hydrocarbon profile changes in the ant *Myrmicaria eumenoides*. J Chem Ecol.

[38] Giray T, Giovanetti M, West-Eberhard MJ (2005). Juvenile hormone, reproduction, and worker behavior in the neotropical social wasp *Polistes canadensis*. Proc Natl Acad Sci USA.

[39] Shorter JR, Tibbetts EA (2009). The effect of juvenile hormone on temporal polyethism in the paper wasp *Polistes dominulus*. Ins soc.

[40] Nijhout, H. F. *Insect Hormones*. (Princeton University Press, 1994).

[41] Rachinsky A, Strambi C, Strambi A, Hartfelder K (1990). Caste and metamorphosis: hemolymph titers of juvenile hormone and ecdysteroids in last instar honeybee larvae. Gen Comp Endocrinol.

[42] Penick CA, Prager SS, Liebig J (2012). Juvenile hormone induces queen development in late-stage larvae of the ant *Harpegnathos saltator*. J Insect Physiol.

[43] Cnaani J, Robinson GE, Hefetz A (2000). The critical period for caste determination in *Bombus terrestris* and its juvenile hormone correlates. J Comp Physiol A.

[44] Bortolotti L, Duchateau MJ, Sbrenna G (2001). Effect of juvenile hormone on caste determination and colony processes in the bumblebee *Bombus terrestris*. Entomol Exp Appl.

[45] Pinto L, Hartfelder K, Bitondi MG, Simões Z (2002). Ecdysteroid titers in pupae of highly social bees relate to distinct modes of caste development. J Insect Physiol.

[46] Hartfelder KH (1987). Rates of juvenile hormone synthesis control caste differentiation in the stingless bee *Scaptotrigona postica depilis*. Rouxs Arch Dev Biol.

[47] Hartfelder K, Rembold H (1991). Caste-specific modulation of juvenile hormone III content and ecdysteroid titer in postembryonic development of the stingless bee. Scaptotrigona postica depilis. J Comp Physiol B.

[48] Cnaani J, Robinson GE, Hefetz A (2000). The critical period for caste determination in *Bombus terrestris* and its juvenile hormone correlates. J Comp Physiol A.

[49] Montagna TS, Raizer J, Antonialli-Junior WF (2015). Effect of Larval Topical Application of Juvenile Hormone on Caste Determination in the Independent-Founding Eusocial Wasp *Mischocyttarus consimilis* (Hymenoptera: Vespidae). OJAS.

[50] Oi CA (2015). Dual Effect of Wasp Queen Pheromone in Regulating Insect Sociality. Curr Biol.

[51] van Zweden JS, Bonckaert W, Wenseleers T, d’Ettorre P (2014). Queen Signaling in Social Wasps. Evolution.

[52] Bonckaert W (2008). Worker policing in the German wasp Vespula germanica. Behav Ecol.

[53] Bonckaert W, Drijfhout FP, d’Ettorre P, Billen J, Wenseleers T (2012). Hydrocarbon Signatures of Egg Maternity, Caste Membership and Reproductive Status in the Common Wasp. J Chem Ecol.

[54] Devillers, J. Juvenile hormones and juvenoids: modeling biologial effects and environmental fate (CRC Press, Boca Raton, 2013).

[55] Haunerland NH, Bowers WS (1985). Comparative studies on pharmacokinetics and metabolism of the anti‐juvenile hormone precocene II. Arch Insect Biochem Physiol.

[56] Agrahari M, Gadagkar R (2003). Juvenile hormone accelerates ovarian development and does not affect age polyethism in the primitively eusocial wasp, *Ropalidia marginata*. J Insect Physiol.

[57] Foster KR, Ratnieks FLW (2001). Convergent evolution of worker policing by egg eating in the honeybee and common wasp. Proc. R. Soc. London, Ser. B.

[58] Helanterä H, Tofilski A, Wenseleers T, Ratnieks FLW (2006). Worker policing in the common wasp *Vespula vulgaris* is not aimed at improving colony hygiene. Ins soc.

[59] Ratnieks FLW, Wenseleers T (2008). Altruism in insect societies and beyond: voluntary or enforced?. Trends Ecol Evol.

[60] Rachinsky A, Hartfelder K (1990). Corpora Allata Activity, a Prime Regulating Element for Caste-Specific Juvenile-Hormone Titer in Honey-Bee Larvae (*Apis Mellifera Carnica*). J Insect Physiol.

[61] Hartfelder K, Engels W (1998). Social insect polymorphism: Hormonal regulation of plasticity in development and reproduction in the honeybee. Curr Top Dev Biol.

[62] Robinson GE, Strambi C, Strambi A, Feldlaufer MF (1991). Comparison of juvenile hormone and ecdysteroid haemolymph titres in adult worker and queen honey bees (*Apis mellifera*). J Insect Physiol.

[63] Ratnieks FLW, Foster KR, Wenseleers T (2006). Conflict resolution in insect societies. Annu Rev Entomol.

[64] Barron AB, Oldroyd BP, Ratnieks FLW (2001). Worker reproduction in honey-bees (*Apis*) and the anarchic syndrome: a review. Behav Ecol Sociobiol.

[65] Wenseleers T, Hart AG, Ratnieks FLW (2004). When resistance is useless: Policing and the evolution of reproductive acquiescence in insect societies. Am Nat.

[66] Wenseleers T, Helantera H, Hart A, Ratnieks FLW (2004). Worker reproduction and policing in insect societies: an ESS analysis. J Evol Biol.

[67] Wenseleers T, Ratnieks FLW (2006). Enforced altruism in insect societies. Nature.

[68] Izzo A, Wells M, Huang Z, Tibbetts E (2010). Cuticular hydrocarbons correlate with fertility, not dominance, in a paper wasp, *Polistes dominulus*. Behav Ecol Sociobiol.

[69] Davey K, Sevala V, Gordon D (1993). The action of juvenile hormone and antigonadotropin on the follicle cells of *Locusta migratoria*. Invertebrate reproduction & development.

[70] Ramaswamy SB, Shu S, Park YI, Zeng F (1997). Dynamics of juvenile hormone‐mediated gonadotropism in the Lepidoptera. Arch Insect Biochem Physiol.

[71] Wicker C, Jallon J (1995). Hormonal control of sex pheromone biosynthesis in *Drosophila melanogaster*. J Insect Physiol.

[72] Trabalon M (1990). Relationships among hormonal changes, cuticular hydrocarbons, and attractiveness during the first gonadotropic cycle of the female *Calliphora vomitoria* (Diptera). Gen Comp Endocrinol.

[73] Howard RW, Blomquist GJ (2005). Ecological, behavioral, andbiochemical aspects of insect hydrocarbons. Annu Rev Entomol.

[74] Morgan, E. D. *Biosynthesis in insects*. (Royal Society of Chemistry, 2004).

[75] Malka O, Katzav-Gozansky T, Hefetz A (2009). Uncoupling fertility from fertility-associated pheromones in worker honeybees (*Apis mellifera*). J Insect Physiol.

[76] Fluri P (1983). Precocene-Ii Has No Anti-Juvenile Hormone Effects in Adult Honey Bees. Experientia.

[77] R: A language and environment for statistical computing (R Foundation for Statistical Computing, Vienna, Austria, 2016).

[78] Messadi D, Helaimia F, Ali-Mokhnache S, Boumahraz M (1990). Accurate determination of retention indices in programmed temperature gas chromatography. Chromatographia.

[79] Aitchison J (1982). The Statistical-Analysis of Compositional Data. Journal of the Royal Statistical Society Series B-Methodological.

[80] Smith, A. A., Millar, J. G. & Suarez, A. V. A social insect fertility signal is dependent on chemical context. *Biol Lett* **11** (2015). 10.1098/rsbl.2014.0947 PMC4321158 25609832

[81] Bretz, F., Hothorn, T. & Westfall, P. *Multiple comparisons using R*. (CRC Press, 2010).

[82] Benjamini, Y. & Yekutieli, D. The control of the false discovery rate in multiple testing under dependency. *Ann Stat* **29**, 1165–1188 (2001).

[83] Kolde, R. Pheatmap: pretty heatmaps. *R package version* **61** (2012).

[84] Lê S, Josse J, Husson F (2008). FactoMineR: an R package for multivariate analysis. Journal of statistical software.

[85] Ripley, B. *et al*. Package ‘MASS’. CRAN Repository. http://cran.r-project.org/web/packages/MASS/MASS.pdf (2013).

[86] Duchateau MJ, Velthuis HHW (1989). Ovarian development and egg laying in workers of *Bombus terrestris*. Entomol Exp Appl.

[87] Brent CS, Vargo EL (2003). Changes in juvenile hormone biosynthetic rate and whole body content in maturing virgin queens of *Solenopsis invicta*. J Insect Physiol.

