# Supplementary material for: Hormonal pleiotropy helps maintain queen signal honesty in a highly eusocial wasp
Source: Sci Rep. 2017 May 10;7:1654. doi: 10.1038/s41598-017-01794-1 (PMC5431770; doi:10.1038/s41598-017-01794-1)
Supplement: Supplementary file 1 — Supplemental material [file 41598_2017_1794_MOESM1_ESM.pdf]

Hormonal pleiotropy helps maintain queen signal honesty in a highly eusocial wasp

Ricardo Caliari Oliveira[1,Ŧ*]; Ayrton Vollet-Neto[1,2,Ŧ]; Cintia Akemi Oi[1]; Jelle S. van Zweden[1];

Fabio Nascimento[2]; Colin Sullivan Brent[3]; Tom Wenseleers[1]

[1] Laboratory of Socioecology and Social Evolution, Zoological Institute, KU Leuven, Leuven, Belgium

[2] Departamento de Biologia, Faculdade de Filosofia, Ciências e Letras de Ribeirão Preto, Universidade de São Paulo, Ribeirão Preto, SP, Brazil

[3] Arid Land Agricultural Research Center, USDA-ARS, Maricopa, Arizona, U.S.A.

[Ŧ] these authors contributed equally

* Corresponding author: ricaliari@gmail.com

Table S1. Absolute abundances in nanograms of CHCs present in *Vespula vulgaris* with 95% confidence intervals of the t distribution.

Compound	Acetone undeveloped			Acetone developed			Precocene undeveloped			Precocene developed		
	Mean	Lower CI	Upper CI	Mean	Lower CI	Upper CI	Mean	Lower CI	Upper CI	Mean	Lower CI	Upper CI
***n*-C21**	12.1	9.5	15.4	10.4	8.6	12.7	16.7	10.2	27.3	10.1	7.9	12.8
***n*-C22**	47.3	38.2	58.6	48.3	41.2	56.5	65.6	49.9	86.1	40.6	33.6	49.2
***n*-C23**	452.9	362.2	567.5	410.2	350.8	479.7	616.6	442.6	859.0	391.7	312.6	492.0
**Mix of 9+11-MeC23**	144.2	101.9	204.6	71.3	55.8	91.0	253.5	147.2	436.5	97.7	73.6	130.0
**7-MeC23**	36.2	24.3	54.1	16.8	12.9	21.8	69.8	37.6	129.4	23.6	17.4	31.9
**5-MeC23**	69.0	47.3	100.7	39.7	31.6	50.1	110.7	63.1	194.5	46.3	35.0	61.5
**3-MeC23**	258.8	203.2	330.4	174.6	145.5	208.9	377.6	257.0	553.4	210.4	169.4	261.2
**5,9-diMeC23**	50.1	35.6	70.5	30.5	24.4	38.1	72.1	41.9	124.2	37.8	27.7	51.4
***n*-C24**	385.5	309.0	479.7	364.8	313.3	423.6	514.0	372.4	709.6	372.4	299.2	463.4
**Mix of 3,9+3,7-diMeC23**	140.9	103.8	191.4	87.7	71.8	107.4	210.9	133.4	333.4	111.7	84.5	147.2
**Mix of 8+9+10+11+12-MeC24**	274.2	198.2	378.4	153.5	123.0	191.4	422.7	255.9	698.2	212.3	160.7	280.5
**6-MeC24**	30.3	21.1	43.7	17.0	13.7	21.1	48.6	27.0	87.5	23.9	18.2	31.4
**4-MeC24**	146.6	110.9	193.6	100.2	83.4	120.5	197.2	126.2	308.3	130.6	102.3	167.1
**C25:1**	131.5	97.1	178.2	83.0	67.5	101.9	219.3	127.9	375.8	113.2	86.7	147.9
**C25:1.y**	51.3	37.2	70.6	33.0	26.6	41.0	78.7	45.7	135.5	42.6	32.4	55.8
**4,8-diMeC24**	153.1	109.6	213.8	91.4	73.6	113.5	221.3	131.8	371.5	129.7	98.2	171.0
***n*-C25**	4092.6	3419.8	4897.8	4477.1	3971.9	5046.6	4487.5	3499.5	5754.4	4508.2	3793.1	5358.0
**Mix of 11+13+15-MeC25**	3630.8	2924.2	4508.2	2691.5	2301.4	3147.7	4315.2	3097.4	5997.9	3349.7	2735.3	4102.0
**7-MeC25**	431.5	319.2	582.1	287.1	237.7	346.7	584.8	367.3	931.1	388.2	300.6	502.3
**5-MeC25**	914.1	712.9	1172.2	698.2	592.9	822.2	1096.5	746.4	1610.6	893.3	716.1	1111.7
**3-MeC25**	2103.8	1749.8	2529.3	2018.4	1786.5	2285.6	2333.5	1745.8	3111.7	2202.9	1853.5	2624.2
**5,13-diMeC25**	826.0	642.7	1059.3	618.0	518.8	737.9	979.5	665.3	1442.1	820.4	639.7	1052.0
***n*-C26**	861.0	696.6	1066.6	1023.3	895.4	1166.8	924.7	706.3	1210.6	1039.9	849.2	1270.6
**Mix of 3,9+3,11-diMeC25**	1370.9	1069.1	1757.9	1044.7	881.0	1235.9	1652.0	1148.2	2376.8	1358.3	1064.1	1737.8
**12-MeC26**	1061.7	849.2	1327.4	903.6	776.2	1054.4	1270.6	901.6	1786.5	1183.0	937.6	1496.2
**6-Mec26**	85.5	64.7	113.0	69.0	57.9	82.4	105.0	67.1	164.1	95.5	72.6	125.6
**4-MeC26**	267.9	209.9	342.8	256.4	219.3	299.2	328.9	227.5	475.3	314.8	246.6	402.7

**C27:1**	514.0	389.0	679.2	485.3	408.3	576.8	648.6	412.1	1020.9	628.1	478.6	824.1
**C27:1.y**	87.3	65.9	115.6	84.9	70.5	102.1	101.6	67.9	152.4	98.9	74.3	131.8
**Mix of4,8+4,6-diMeC26**	298.5	228.6	389.0	233.3	195.9	278.0	380.2	261.2	554.6	337.3	261.2	435.5
***n*-C27**	3953.7	3155.0	4965.9	5345.6	4698.9	6081.4	3723.9	2930.9	4742.4	5211.9	4335.1	6251.7
**Mix of 9+11+13-MeC27**	3639.2	2924.2	4529.0	3732.5	3281.0	4256.0	3854.8	2904.0	5116.8	4375.2	3548.1	5407.5
**7-MeC27**	239.3	187.1	306.9	272.9	233.9	317.7	246.6	169.8	357.3	312.6	243.2	401.8
**5-MeC27**	343.6	254.1	464.5	362.2	306.9	426.6	383.7	267.9	549.5	408.3	304.8	547.0
**11,15-diMeC27**	310.5	250.0	385.5	290.4	252.3	333.4	393.6	294.4	524.8	360.6	290.4	448.7
**3-MeC27**	1757.9	1393.2	2223.3	2466.0	2147.8	2831.4	1659.6	1247.4	2208.0	2371.4	1967.9	2851.0
**5,13-diMeC27**	676.1	523.6	875.0	679.2	584.8	790.7	776.2	570.2	1054.4	869.0	671.4	1127.2
***n*-C28**	333.4	258.2	430.5	467.7	402.7	544.5	310.5	242.7	397.2	455.0	368.1	561.0
**Mix of 3,11+3,13- diMeC27**	1798.9	1435.5	2249.1	1733.8	1513.6	1986.1	1967.9	1465.5	2648.5	2259.4	1823.9	2792.5
**Mix of 10+12+14+16-MeC28**	456.0	342.0	606.7	511.7	434.5	602.6	450.8	326.6	622.3	623.7	475.3	816.6
**4-MeC28**	154.9	116.7	205.6	194.5	164.4	230.1	171.8	123.3	238.8	213.3	164.8	276.1
**C29:1**	261.2	205.6	331.9	358.9	306.2	421.7	263.0	181.6	380.2	351.6	281.8	439.5
**Mix of 4,12+4,14-diMeC28**	233.9	175.0	311.9	242.1	203.7	287.1	254.1	180.3	357.3	317.7	237.7	423.6
***n*-C29**	645.7	474.2	879.0	1069.1	903.6	1267.7	495.5	377.6	650.1	975.0	765.6	1241.7
**Mix of 11+13+15-MeC29**	701.5	515.2	955.0	1069.1	897.4	1276.4	590.2	432.5	805.4	1042.3	785.2	1380.4
**7-MeC29**	38.6	29.5	50.6	72.8	59.2	89.5	29.3	21.9	39.4	57.8	44.3	75.7
**5-MeC29**	45.5	30.8	67.1	69.5	56.0	86.3	41.8	29.1	60.0	64.9	45.1	93.1
**Mix of 9,19+9,15-diMeC29**	113.5	84.7	151.7	187.9	152.1	232.3	111.9	83.2	150.3	184.1	136.8	247.2
**3-MeC29**	252.9	183.7	348.3	548.3	438.5	685.5	191.9	140.3	263.0	418.8	318.4	549.5
**5,9-diMeC29**	320.6	233.3	440.6	407.4	342.8	485.3	315.5	234.4	424.6	448.7	336.5	597.0
***n*-C30**	410.2	311.9	539.5	503.5	430.5	587.5	402.7	305.5	530.9	548.3	424.6	707.9
**Mix of10+11+12+13+14-MeC30**	63.8	47.4	85.9	109.1	89.3	133.4	52.8	39.6	70.5	97.3	72.8	130.0
**C31:1**	6.0	4.4	8.0	11.4	9.1	14.3	4.9	3.1	7.7	8.6	6.4	11.5
***n*-C31**	6.3	4.3	9.4	11.4	9.2	14.1	4.4	3.0	6.5	9.4	6.9	12.7
**Mix of 11+13+15-MeC31**	23.9	16.3	34.8	56.6	44.1	72.6	15.8	10.6	23.7	39.8	28.2	56.2
**Mix of 11,17+13,17+15,19-diMeC31**	56.5	41.1	77.6	146.9	107.6	200.4	39.2	26.7	57.5	95.9	65.2	140.9
**3-MeC31**	71.4	50.1	102.1	156.7	124.2	197.2	51.9	36.3	74.0	110.7	84.3	145.5
**5,13-diMeC31**	83.2	61.9	111.7	126.2	105.4	151.0	74.0	55.2	99.1	105.4	83.2	134.0
**13+15-MeC33**	15.7	11.3	21.9	29.1	23.6	36.0	11.8	8.7	16.1	24.1	18.2	31.8

**11,21-diMeC33**	98.4	70.0	138.4	249.5	178.6	347.5	61.4	42.5	88.7	149.3	100.0	222.8

Table S1 continued. Absolute abundances in nanograms of CHCs present in *Vespula vulgaris* with 95% confidence intervals of the t distribution.

Compound	Methoprene undeveloped			Methoprene developed			Queens		
	Mean	Lower CI	Upper CI	Mean	Lower CI	Upper CI	Mean	Lower CI	Upper CI
***n*-C21**	10.2	5.7	18.3	8.5	7.3	9.9	9.3	6.0	14.6
***n*-C22**	55.6	36.1	85.7	42.0	35.4	49.9	47.2	24.3	91.8
***n*-C23**	385.5	240.4	618.0	307.6	264.2	358.9	521.2	304.8	889.2
**Mix of 9+11-MeC23**	47.3	26.4	84.7	30.8	24.0	39.5	80.0	39.9	160.0
**7-MeC23**	10.0	5.4	18.3	6.7	5.2	8.5	19.1	10.0	36.1
**5-MeC23**	29.2	16.3	52.5	22.4	17.9	28.2	39.1	20.6	74.5
**3-MeC23**	150.7	92.9	243.8	108.1	86.3	135.8	169.4	78.9	363.1
**5,9-diMeC23**	24.4	14.4	41.4	19.9	16.3	24.2	28.1	14.6	54.0
***n*-C24**	318.4	211.3	479.7	282.5	242.1	329.6	597.0	369.0	966.1
**Mix of 3,9+3,7-diMeC23**	68.4	43.6	107.2	52.2	43.3	63.1	100.0	53.8	185.8
**Mix of 8+9+10+11+12-MeC24**	103.3	61.4	174.2	75.3	60.8	93.1	108.1	54.5	214.3
**6-MeC24**	10.9	6.6	18.2	9.1	7.6	10.9	11.9	6.2	22.9
**4-MeC24**	73.1	45.5	117.8	59.0	48.6	71.6	95.5	47.8	191.0
**C25:1**	73.1	42.2	127.1	55.1	41.0	74.0	129.7	72.3	233.3
**C25:1.y**	26.0	14.0	48.1	19.9	15.2	26.0	45.4	25.9	79.6
**4,8-diMeC24**	61.4	35.7	105.7	45.7	36.6	57.0	80.7	42.5	153.5
***n*-C25**	4265.8	3047.9	5984.1	4140.0	3639.2	4698.9	21037.8	15417.0	28774.0
**Mix of 11+13+15-MeC25**	2027.7	1374.0	2992.3	1552.4	1297.2	1857.8	2582.3	1538.2	4335.1
**7-MeC25**	190.1	115.6	311.9	147.6	119.1	182.8	219.8	132.7	364.8
**5-MeC25**	469.9	299.9	734.5	389.0	323.6	467.7	545.8	327.3	909.9
**3-MeC25**	1963.4	1432.2	2691.5	1725.8	1517.1	1967.9	3054.9	1866.4	4988.8
**5,13-diMeC25**	450.8	293.8	691.8	349.1	289.1	421.7	503.5	295.1	857.0

***n*-C26**	999.1	690.2	1448.8	1037.5	885.1	1219.0	5546.3	3819.4	8053.8
**Mix of 3,9+3,11-diMeC25**	811.0	550.8	1194.0	647.1	544.5	769.1	1135.0	693.4	1857.8
**12-MeC26**	638.3	433.5	939.7	548.3	469.9	639.7	514.0	289.7	912.0
**6-Mec26**	44.5	28.2	70.0	40.1	33.7	47.8	30.5	16.4	56.8
**4-MeC26**	200.9	135.5	297.2	189.2	161.1	222.3	214.8	119.1	386.4
**C27:1**	437.5	272.9	699.8	400.9	329.6	488.7	635.3	406.4	993.1
**C27:1.y**	63.8	36.3	112.2	54.5	42.5	69.7	95.9	55.1	167.5
**Mix of4,8+4,6-diMeC26**	147.6	96.2	227.0	119.4	100.2	142.2	100.5	50.2	200.9
***n*-C27**	5714.8	4045.8	8053.8	6208.7	5407.5	7112.1	38282.5	29040.2	50466.1
**Mix of 9+11+13-MeC27**	2884.0	2147.8	3863.7	2729.0	2426.6	3076.1	2546.8	1610.6	4027.2
**7-MeC27**	230.1	158.9	332.7	247.7	215.8	285.1	309.0	189.7	502.3
**5-MeC27**	274.2	195.4	385.5	259.4	224.9	298.5	236.0	148.3	375.8
**11,15-diMeC27**	204.2	143.5	290.4	201.8	173.0	235.5	209.9	129.1	342.0
**3-MeC27**	2824.9	2055.9	3881.5	3133.3	2722.7	3605.8	7620.8	5272.3	11015.4
**5,13-diMeC27**	553.4	396.3	774.5	503.5	434.5	584.8	597.0	409.3	873.0
***n*-C28**	518.8	341.2	790.7	610.9	510.5	729.5	1972.4	1358.3	2864.2
**Mix of 3,11+3,13- diMeC27**	1330.5	972.7	1823.9	1199.5	1069.1	1349.0	1318.3	849.2	2051.2
**Mix of 10+12+14+16-MeC28**	419.8	298.5	588.8	428.5	366.4	500.0	281.2	152.8	517.6
**4-MeC28**	179.5	119.7	268.5	206.5	169.8	250.6	222.8	136.1	364.8
**C29:1**	353.2	233.3	534.6	410.2	356.5	473.2	765.6	511.7	1145.5
**Mix of 4,12+4,14-diMeC28**	176.2	121.9	254.1	163.3	139.6	190.5	218.8	123.0	389.0
***n*-C29**	1412.5	905.7	2208.0	1694.3	1412.5	2037.0	8851.2	6471.4	12106.0
**Mix of 11+13+15-MeC29**	1047.1	755.1	1455.5	1219.0	1039.9	1425.6	869.0	587.5	1282.3
**7-MeC29**	105.0	71.3	154.9	152.4	125.9	184.1	140.0	98.2	199.5
**5-MeC29**	70.1	47.3	103.8	83.0	68.4	100.9	116.1	84.3	160.3
**Mix of 9,19+9,15-diMeC29**	216.3	135.5	344.3	293.1	234.4	367.3	287.1	192.8	427.6
**3-MeC29**	877.0	558.5	1374.0	1188.5	961.6	1468.9	4720.6	3372.9	6606.9
**5,9-diMeC29**	359.7	257.0	504.7	376.7	318.4	445.7	787.0	595.7	1042.3
***n*-C30**	430.5	299.9	616.6	431.5	372.4	500.0	576.8	402.7	826.0
**Mix of10+11+12+13+14-MeC30**	118.3	80.4	173.8	159.2	131.2	193.6	64.7	41.2	101.6
**C31:1**	13.6	8.3	22.3	18.8	15.2	23.2	32.7	20.1	53.0
***n*-C31**	13.7	7.0	26.7	15.5	12.3	19.5	76.9	45.1	131.2

**Mix of 11+13+15-MeC31**	75.0	48.1	116.7	103.3	83.8	127.6	42.0	25.9	67.9
**Mix of 11,17+13,17+15,19-diMeC31**	339.6	190.5	605.3	576.8	436.5	762.1	542.0	379.3	772.7
**3-MeC31**	231.2	145.9	365.6	310.5	248.3	388.2	1101.5	717.8	1690.4
**5,13-diMeC31**	119.9	83.4	173.0	123.3	105.7	143.5	116.7	80.0	170.2
**13+15-MeC33**	33.3	22.1	50.4	44.4	35.6	55.1	21.8	14.9	31.8
**11,21-diMeC33**	691.8	378.4	1264.7	1221.8	937.6	1595.9	1496.2	1061.7	2108.6

Table S2. Treatment - Control contrasts of Aitchison transformed CHC based on linear mixed models with p values fdr corrected according to Benjamini and Yekutieli.

Compound	Contrasts	Coefficients	t.ratio	p.value	padj	Significance level
Mix of10+11+12+13+14-MeC30	Acetone.undeveloped - Methoprene.undeveloped	-0.674	-3.983	0.0001	0.004	* *
Mix of10+11+12+13+14-MeC30	Acetone.undeveloped - Acetone.developed	-0.447	-3.395	0.001	0.021	*
Mix of10+11+12+13+14-MeC30	Acetone.undeveloped - Precocene.undeveloped	0.048	0.372	0.711	1	
Mix of10+11+12+13+14-MeC30	Acetone.developed - Methoprene.developed	-0.541	-4.116	0.0001	0.003	* *
Mix of10+11+12+13+14-MeC30	Acetone.developed - Precocene.developed	-0.125	-1.102	0.272	1	
Mix of10+11+12+13+14-MeC30	Methoprene.developed - Queen.developed	1.454	8.035	0	0.0002	* * *
Mix of 10+12+14+16-MeC28	Acetone.undeveloped - Methoprene.undeveloped	-0.126	-1.286	0.2	1	
Mix of 10+12+14+16-MeC28	Acetone.undeveloped - Acetone.developed	0.043	0.559	0.577	1	
Mix of 10+12+14+16-MeC28	Acetone.undeveloped - Precocene.undeveloped	-0.121	-1.62	0.107	1	
Mix of 10+12+14+16-MeC28	Acetone.developed - Methoprene.developed	-0.236	-3.09	0.002	0.049	*
Mix of 10+12+14+16-MeC28	Acetone.developed - Precocene.developed	-0.303	-4.588	0.00001	0.001	* * *
Mix of 10+12+14+16-MeC28	Methoprene.developed - Queen.developed	1.058	5.967	0.0001	0.003	* *
11,21-diMeC33	Acetone.undeveloped - Methoprene.undeveloped	-1.389	-4.437	0.00002	0.001	* *
11,21-diMeC33	Acetone.undeveloped - Acetone.developed	-0.828	-3.342	0.001	0.023	*
11,21-diMeC33	Acetone.undeveloped - Precocene.undeveloped	0.285	1.179	0.24	1	
11,21-diMeC33	Acetone.developed - Methoprene.developed	-1.12	-4.73	0.00005	0.003	* *
11,21-diMeC33	Acetone.developed - Precocene.developed	0.041	0.195	0.846	1	
11,21-diMeC33	Methoprene.developed - Queen.developed	0.124	0.48	0.634	1	
Mix of 11+13+15-MeC31	Acetone.undeveloped - Methoprene.undeveloped	-0.804	-3.425	0.001	0.019	*
Mix of 11+13+15-MeC31	Acetone.undeveloped - Acetone.developed	-0.652	-3.567	0.0005	0.013	*
Mix of 11+13+15-MeC31	Acetone.undeveloped - Precocene.undeveloped	0.175	0.979	0.329	1	
Mix of 11+13+15-MeC31	Acetone.developed - Methoprene.developed	-0.46	-2.519	0.014	0.197	
Mix of 11+13+15-MeC31	Acetone.developed - Precocene.developed	0.062	0.389	0.698	1	
Mix of 11+13+15-MeC31	Methoprene.developed - Queen.developed	1.317	6.231	0	0.0003	* * *
11,15-diMeC27	Acetone.undeveloped - Methoprene.undeveloped	0.075	0.796	0.427	1	
11,15-diMeC27	Acetone.undeveloped - Acetone.developed	0.065	0.884	0.378	1	
11,15-diMeC27	Acetone.undeveloped - Precocene.undeveloped	-0.08	-1.111	0.268	1	
11,15-diMeC27	Acetone.developed - Methoprene.developed	0.017	0.228	0.82	1	

11,15-diMeC27	Acetone.developed - Precocene.developed	-0.108	-1.699	0.091	1	
11,15-diMeC27	Methoprene.developed - Queen.developed	0.456	3.629	0.003	0.064	
Mix of 11,17+13,17+15,19-diMeC31	Acetone.undeveloped - Methoprene.undeveloped	-1.396	-4.632	0.00001	0.001	* * *
Mix of 11,17+13,17+15,19-diMeC31	Acetone.undeveloped - Acetone.developed	-0.894	-3.693	0.0003	0.009	* *
Mix of 11,17+13,17+15,19-diMeC31	Acetone.undeveloped - Precocene.undeveloped	0.223	0.948	0.345	1	
Mix of 11,17+13,17+15,19-diMeC31	Acetone.developed - Methoprene.developed	-1.039	-4.692	0.0001	0.005	* *
Mix of 11,17+13,17+15,19-diMeC31	Acetone.developed - Precocene.developed	0.004	0.021	0.984	1	
Mix of 11,17+13,17+15,19-diMeC31	Methoprene.developed - Queen.developed	0.448	1.847	0.072	0.846	
Mix of 11+13+15-MeC25	Acetone.undeveloped - Methoprene.undeveloped	0.554	4.602	0.00001	0.001	* * *
Mix of 11+13+15-MeC25	Acetone.undeveloped - Acetone.developed	0.385	4.119	0.0001	0.003	* *
Mix of 11+13+15-MeC25	Acetone.undeveloped - Precocene.undeveloped	0.072	0.788	0.432	1	
Mix of 11+13+15-MeC25	Acetone.developed - Methoprene.developed	0.413	4.407	0.00002	0.001	* *
Mix of 11+13+15-MeC25	Acetone.developed - Precocene.developed	0.084	1.04	0.3	1	
Mix of 11+13+15-MeC25	Methoprene.developed - Queen.developed	-0.169	-1.446	0.164	1	
Mix of 11+13+15-MeC29	Acetone.undeveloped - Methoprene.undeveloped	-0.555	-4.006	0.0001	0.004	* *
Mix of 11+13+15-MeC29	Acetone.undeveloped - Acetone.developed	-0.25	-2.313	0.022	0.314	
Mix of 11+13+15-MeC29	Acetone.undeveloped - Precocene.undeveloped	-0.037	-0.348	0.729	1	
Mix of 11+13+15-MeC29	Acetone.developed - Methoprene.developed	-0.483	-4.487	0.00001	0.001	* *
Mix of 11+13+15-MeC29	Acetone.developed - Precocene.developed	-0.187	-2	0.047	0.621	
Mix of 11+13+15-MeC29	Methoprene.developed - Queen.developed	0.977	5.746	0.0001	0.003	* *
12-MeC26	Acetone.undeveloped - Methoprene.undeveloped	0.347	4.416	0.00002	0.001	* *
12-MeC26	Acetone.undeveloped - Acetone.developed	0.176	2.873	0.005	0.085	
12-MeC26	Acetone.undeveloped - Precocene.undeveloped	0.013	0.224	0.823	1	
12-MeC26	Acetone.developed - Methoprene.developed	0.288	4.713	0.00001	0.001	* * *
12-MeC26	Acetone.developed - Precocene.developed	-0.071	-1.344	0.181	1	
12-MeC26	Methoprene.developed - Queen.developed	0.489	3.449	0.005	0.084	
Mix of 13+15-MeC33	Acetone.undeveloped - Methoprene.undeveloped	-0.569	-2.997	0.003	0.061	
Mix of 13+15-MeC34	Acetone.undeveloped - Acetone.developed	-0.409	-2.771	0.006	0.103	
Mix of 13+15-MeC35	Acetone.undeveloped - Precocene.undeveloped	0.107	0.741	0.46	1	
Mix of 13+15-MeC36	Acetone.developed - Methoprene.developed	-0.444	-3.007	0.003	0.064	
Mix of 13+15-MeC37	Acetone.developed - Precocene.developed	-0.034	-0.267	0.79	1	
Mix of 13+15-MeC38	Methoprene.developed - Queen.developed	1.184	6.77	0	0.0002	* * *

3-MeC23	Acetone.undeveloped - Methoprene.undeveloped	0.345	2.136	0.034	0.464	
3-MeC23	Acetone.undeveloped - Acetone.developed	0.401	3.183	0.002	0.038	*
3-MeC23	Acetone.undeveloped - Precocene.undeveloped	-0.099	-0.809	0.42	1	
3-MeC23	Acetone.developed - Methoprene.developed	0.25	1.989	0.048	0.628	
3-MeC23	Acetone.developed - Precocene.developed	0.11	1.012	0.313	1	
3-MeC23	Methoprene.developed - Queen.developed	-0.063	-0.262	0.798	1	
3-MeC25	Acetone.undeveloped - Methoprene.undeveloped	0.043	0.649	0.517	1	
3-MeC25	Acetone.undeveloped - Acetone.developed	0.117	2.277	0.024	0.342	
3-MeC25	Acetone.undeveloped - Precocene.undeveloped	0.065	1.292	0.198	1	
3-MeC25	Acetone.developed - Methoprene.developed	0.03	0.58	0.563	1	
3-MeC25	Acetone.developed - Precocene.developed	0.08	1.79	0.075	0.879	
3-MeC25	Methoprene.developed - Queen.developed	-0.184	-2.468	0.027	0.38	
3-MeC27	Acetone.undeveloped - Methoprene.undeveloped	-0.448	-3.462	0.001	0.018	*
3-MeC27	Acetone.undeveloped - Acetone.developed	-0.225	-2.231	0.027	0.38	
3-MeC27	Acetone.undeveloped - Precocene.undeveloped	0.041	0.421	0.674	1	
3-MeC27	Acetone.developed - Methoprene.developed	-0.36	-3.597	0.001	0.016	*
3-MeC27	Acetone.developed - Precocene.developed	-0.023	-0.262	0.794	1	
3-MeC27	Methoprene.developed - Queen.developed	-0.413	-3.672	0.001	0.022	*
3-MeC29	Acetone.undeveloped - Methoprene.undeveloped	-0.932	-4.248	0.00004	0.002	* *
3-MeC29	Acetone.undeveloped - Acetone.developed	-0.618	-3.519	0.001	0.015	*
3-MeC29	Acetone.undeveloped - Precocene.undeveloped	0.135	0.792	0.429	1	
3-MeC29	Acetone.developed - Methoprene.developed	-0.638	-3.927	0.001	0.018	*
3-MeC29	Acetone.developed - Precocene.developed	-0.005	-0.034	0.973	1	
3-MeC29	Methoprene.developed - Queen.developed	-0.953	-5.373	0	0.001	* * *
3-MeC31	Acetone.undeveloped - Methoprene.undeveloped	-0.864	-3.828	0.0002	0.006	* *
3-MeC31	Acetone.undeveloped - Acetone.developed	-0.601	-3.414	0.001	0.02	*
3-MeC31	Acetone.undeveloped - Precocene.undeveloped	0.176	1.029	0.305	1	
3-MeC31	Acetone.developed - Methoprene.developed	-0.537	-3.074	0.003	0.061	
3-MeC31	Acetone.developed - Precocene.developed	0.091	0.6	0.549	1	
3-MeC31	Methoprene.developed - Queen.developed	-0.877	-4.462	0.0001	0.004	* *
Mix of 3,11+3,13- diMeC27	Acetone.undeveloped - Methoprene.undeveloped	0.055	0.791	0.43	1	
Mix of 3,11+3,13- diMeC27	Acetone.undeveloped - Acetone.developed	0.102	1.879	0.062	0.748	

Mix of 3,11+3,13- diMeC27	Acetone.undeveloped - Precocene.undeveloped	-0.017	-0.324	0.746	1	
Mix of 3,11+3,13- diMeC27	Acetone.developed - Methoprene.developed	0.005	0.084	0.933	1	
Mix of 3,11+3,13- diMeC27	Acetone.developed - Precocene.developed	-0.169	-3.598	0.0004	0.012	*
Mix of 3,11+3,13- diMeC27	Methoprene.developed - Queen.developed	0.447	4.347	0.001	0.022	*
Mix of 3,9+3,11-diMeC25	Acetone.undeveloped - Methoprene.undeveloped	0.535	4.385	0.00002	0.001	* *
Mix of 3,9+3,11-diMeC25	Acetone.undeveloped - Acetone.developed	0.269	2.838	0.005	0.093	
Mix of 3,9+3,11-diMeC25	Acetone.undeveloped - Precocene.undeveloped	0.165	1.778	0.077	0.893	
Mix of 3,9+3,11-diMeC25	Acetone.developed - Methoprene.developed	0.467	4.926	0	0.0004	* * *
Mix of 3,9+3,11-diMeC25	Acetone.developed - Precocene.developed	0.098	1.198	0.233	1	
Mix of 3,9+3,11-diMeC25	Methoprene.developed - Queen.developed	-0.3	-2.168	0.048	0.628	
Mix of 3,9+3,7-diMeC23	Acetone.undeveloped - Methoprene.undeveloped	0.715	4.166	0.0001	0.003	* *
Mix of 3,9+3,7-diMeC23	Acetone.undeveloped - Acetone.developed	0.409	3.062	0.003	0.052	
Mix of 3,9+3,7-diMeC23	Acetone.undeveloped - Precocene.undeveloped	0.057	0.437	0.663	1	
Mix of 3,9+3,7-diMeC23	Acetone.developed - Methoprene.developed	0.54	4.055	0.0001	0.003	* *
Mix of 3,9+3,7-diMeC23	Acetone.developed - Precocene.developed	0.215	1.864	0.064	0.765	
Mix of 3,9+3,7-diMeC23	Methoprene.developed - Queen.developed	-0.432	-2.141	0.052	0.661	
4-MeC24	Acetone.undeveloped - Methoprene.undeveloped	0.686	4.599	0.00001	0.001	* * *
4-MeC24	Acetone.undeveloped - Acetone.developed	0.302	2.6	0.01	0.154	
4-MeC24	Acetone.undeveloped - Precocene.undeveloped	0.121	1.066	0.288	1	
4-MeC24	Acetone.developed - Methoprene.developed	0.572	4.943	0	0.0004	* * *
4-MeC24	Acetone.developed - Precocene.developed	0.147	1.46	0.146	1	
4-MeC24	Methoprene.developed - Queen.developed	-0.258	-1.211	0.249	1	
4-MeC26	Acetone.undeveloped - Methoprene.undeveloped	0.073	1.208	0.229	1	
4-MeC26	Acetone.undeveloped - Acetone.developed	-0.014	-0.29	0.772	1	
4-MeC26	Acetone.undeveloped - Precocene.undeveloped	-0.028	-0.614	0.54	1	
4-MeC26	Acetone.developed - Methoprene.developed	0.093	1.97	0.051	0.655	
4-MeC26	Acetone.developed - Precocene.developed	-0.106	-2.599	0.01	0.154	
4-MeC26	Methoprene.developed - Queen.developed	0.343	2.025	0.062	0.748	
4-MeC28	Acetone.undeveloped - Methoprene.undeveloped	-0.464	-3.593	0.0004	0.012	*
4-MeC28	Acetone.undeveloped - Acetone.developed	-0.261	-2.597	0.01	0.154	
4-MeC28	Acetone.undeveloped - Precocene.undeveloped	-0.12	-1.228	0.221	1	
4-MeC28	Acetone.developed - Methoprene.developed	-0.419	-4.186	0.00005	0.002	* *

4-MeC28	Acetone.developed - Precocene.developed	-0.242	-2.788	0.006	0.1	
4-MeC28	Methoprene.developed - Queen.developed	0.568	3.341	0.006	0.098	
Mix of 4,12+4,14-diMeC28	Acetone.undeveloped - Methoprene.undeveloped	-0.095	-1.022	0.308	1	
Mix of 4,12+4,14-diMeC28	Acetone.undeveloped - Acetone.developed	-0.02	-0.272	0.786	1	
Mix of 4,12+4,14-diMeC28	Acetone.undeveloped - Precocene.undeveloped	-0.128	-1.814	0.072	0.845	
Mix of 4,12+4,14-diMeC28	Acetone.developed - Methoprene.developed	-0.065	-0.9	0.37	1	
Mix of 4,12+4,14-diMeC28	Acetone.developed - Precocene.developed	-0.326	-5.205	0	0.0002	* * *
Mix of 4,12+4,14-diMeC28	Methoprene.developed - Queen.developed	0.361	1.842	0.088	1	
4,8-diMeC24	Acetone.undeveloped - Methoprene.undeveloped	0.923	4.789	0	0.001	* * *
4,8-diMeC24	Acetone.undeveloped - Acetone.developed	0.419	2.794	0.006	0.1	
4,8-diMeC24	Acetone.undeveloped - Precocene.undeveloped	0.084	0.575	0.566	1	
4,8-diMeC24	Acetone.developed - Methoprene.developed	0.76	5.072	0	0.0003	* * *
4,8-diMeC24	Acetone.developed - Precocene.developed	0.134	1.033	0.303	1	
4,8-diMeC24	Methoprene.developed - Queen.developed	-0.371	-1.719	0.108	1	
Mix of4,8+4,6-diMeC26	Acetone.undeveloped - Methoprene.undeveloped	0.528	4.91	0	0.0004	* * *
Mix of4,8+4,6-diMeC26	Acetone.undeveloped - Acetone.developed	0.163	1.95	0.053	0.677	
Mix of4,8+4,6-diMeC26	Acetone.undeveloped - Precocene.undeveloped	0.052	0.633	0.528	1	
Mix of4,8+4,6-diMeC26	Acetone.developed - Methoprene.developed	0.538	6.441	0	0	* * *
Mix of4,8+4,6-diMeC26	Acetone.developed - Precocene.developed	-0.076	-1.056	0.293	1	
Mix of4,8+4,6-diMeC26	Methoprene.developed - Queen.developed	0.531	2.211	0.045	0.598	
5-MeC23	Acetone.undeveloped - Methoprene.undeveloped	0.947	4.151	0.0001	0.003	* *
5-MeC23	Acetone.undeveloped - Acetone.developed	0.482	2.711	0.007	0.119	
5-MeC23	Acetone.undeveloped - Precocene.undeveloped	0.038	0.221	0.826	1	
5-MeC23	Acetone.developed - Methoprene.developed	0.69	3.893	0.0001	0.005	* *
5-MeC23	Acetone.developed - Precocene.developed	0.384	2.499	0.013	0.197	
5-MeC23	Methoprene.developed - Queen.developed	-0.407	-1.578	0.137	1	
5-MeC25	Acetone.undeveloped - Methoprene.undeveloped	0.648	5.218	0	0.0002	* * *
5-MeC25	Acetone.undeveloped - Acetone.developed	0.3	3.106	0.002	0.047	*
5-MeC25	Acetone.undeveloped - Precocene.undeveloped	0.08	0.853	0.395	1	
5-MeC25	Acetone.developed - Methoprene.developed	0.498	5.161	0	0.0002	* * *
5-MeC25	Acetone.developed - Precocene.developed	0.055	0.654	0.514	1	
5-MeC25	Methoprene.developed - Queen.developed	-0.008	-0.06	0.953	1	

5-MeC27	Acetone.undeveloped - Methoprene.undeveloped	-0.126	-1.221	0.224	1	
5-MeC27	Acetone.undeveloped - Acetone.developed	0.043	0.535	0.593	1	
5-MeC27	Acetone.undeveloped - Precocene.undeveloped	-0.215	-2.755	0.007	0.107	
5-MeC27	Acetone.developed - Methoprene.developed	-0.21	-2.631	0.009	0.143	
5-MeC27	Acetone.developed - Precocene.developed	-0.234	-3.38	0.001	0.022	*
5-MeC27	Methoprene.developed - Queen.developed	0.825	5.073	0.0003	0.008	* *
5-MeC29	Acetone.undeveloped - Methoprene.undeveloped	-0.701	-3.91	0.0001	0.005	* *
5-MeC29	Acetone.undeveloped - Acetone.developed	-0.308	-2.207	0.029	0.396	
5-MeC29	Acetone.undeveloped - Precocene.undeveloped	-0.208	-1.53	0.128	1	
5-MeC29	Acetone.developed - Methoprene.developed	-0.652	-4.684	0.00001	0.001	* * *
5-MeC29	Acetone.developed - Precocene.developed	-0.332	-2.75	0.007	0.107	
5-MeC29	Methoprene.developed - Queen.developed	0.463	2.087	0.058	0.712	
5,13-diMeC25	Acetone.undeveloped - Methoprene.undeveloped	0.637	4.898	0	0.0004	* * *
5,13-diMeC25	Acetone.undeveloped - Acetone.developed	0.273	2.7	0.008	0.122	
5,13-diMeC25	Acetone.undeveloped - Precocene.undeveloped	0.181	1.838	0.068	0.806	
5,13-diMeC25	Acetone.developed - Methoprene.developed	0.597	5.916	0	0.00002	* * *
5,13-diMeC25	Acetone.developed - Precocene.developed	0.088	1.01	0.314	1	
5,13-diMeC25	Methoprene.developed - Queen.developed	-0.123	-0.824	0.424	1	
5,13-diMeC27	Acetone.undeveloped - Methoprene.undeveloped	-0.154	-1.889	0.061	0.738	
5,13-diMeC27	Acetone.undeveloped - Acetone.developed	0.041	0.642	0.522	1	
5,13-diMeC27	Acetone.undeveloped - Precocene.undeveloped	-0.137	-2.206	0.029	0.396	
5,13-diMeC27	Acetone.developed - Methoprene.developed	-0.174	-2.748	0.007	0.107	
5,13-diMeC27	Acetone.developed - Precocene.developed	-0.262	-4.758	0	0.001	* * *
5,13-diMeC27	Methoprene.developed - Queen.developed	0.477	3.291	0.006	0.102	
5,13-diMeC31	Acetone.undeveloped - Methoprene.undeveloped	-0.292	-2.144	0.034	0.457	
5,13-diMeC31	Acetone.undeveloped - Acetone.developed	-0.234	-2.212	0.028	0.395	
5,13-diMeC31	Acetone.undeveloped - Precocene.undeveloped	0.06	0.584	0.56	1	
5,13-diMeC31	Acetone.developed - Methoprene.developed	-0.09	-0.845	0.4	1	
5,13-diMeC31	Acetone.developed - Precocene.developed	0.1	1.096	0.275	1	
5,13-diMeC31	Methoprene.developed - Queen.developed	0.514	4.046	0.001	0.014	*
5,9-diMeC29	Acetone.undeveloped - Methoprene.undeveloped	-0.48	-3.584	0.0005	0.013	*
5,9-diMeC29	Acetone.undeveloped - Acetone.developed	-0.124	-1.185	0.238	1	

5,9-diMeC29	Acetone.undeveloped - Precocene.undeveloped	-0.136	-1.34	0.182	1	
5,9-diMeC29	Acetone.developed - Methoprene.developed	-0.454	-4.368	0.00002	0.001	* *
5,9-diMeC29	Acetone.developed - Precocene.developed	-0.254	-2.818	0.005	0.095	
5,9-diMeC29	Methoprene.developed - Queen.developed	-0.022	-0.112	0.912	1	
5_9-diMeC23	Acetone.undeveloped - Methoprene.undeveloped	0.846	4.109	0.0001	0.003	* *
5_9-diMeC23	Acetone.undeveloped - Acetone.developed	0.431	2.686	0.008	0.125	
5_9-diMeC23	Acetone.undeveloped - Precocene.undeveloped	0.099	0.635	0.526	1	
5_9-diMeC23	Acetone.developed - Methoprene.developed	0.582	3.637	0.0004	0.011	*
5_9-diMeC23	Acetone.developed - Precocene.developed	0.244	1.759	0.081	0.92	
5_9-diMeC23	Methoprene.developed - Queen.developed	-0.183	-0.745	0.47	1	
6-MeC24	Acetone.undeveloped - Methoprene.undeveloped	0.851	4.138	0.0001	0.003	* *
6-MeC24	Acetone.undeveloped - Acetone.developed	0.446	2.786	0.006	0.1	
6-MeC24	Acetone.undeveloped - Precocene.undeveloped	-0.053	-0.338	0.736	1	
6-MeC24	Acetone.developed - Methoprene.developed	0.501	3.133	0.002	0.044	*
6-MeC24	Acetone.developed - Precocene.developed	0.054	0.387	0.699	1	
6-MeC24	Methoprene.developed - Queen.developed	0.073	0.337	0.74	1	
6-Mec26	Acetone.undeveloped - Methoprene.undeveloped	0.492	4.087	0.0001	0.003	* *
6-Mec26	Acetone.undeveloped - Acetone.developed	0.131	1.401	0.163	1	
6-Mec26	Acetone.undeveloped - Precocene.undeveloped	0.036	0.397	0.692	1	
6-Mec26	Acetone.developed - Methoprene.developed	0.394	4.217	0.00004	0.002	* *
6-Mec26	Acetone.developed - Precocene.developed	-0.108	-1.329	0.186	1	
6-Mec26	Methoprene.developed - Queen.developed	0.687	4.112	0.001	0.031	*
7-MeC23	Acetone.undeveloped - Methoprene.undeveloped	1.122	4.044	0.0001	0.003	* *
7-MeC23	Acetone.undeveloped - Acetone.developed	0.739	3.424	0.001	0.019	*
7-MeC23	Acetone.undeveloped - Precocene.undeveloped	-0.25	-1.19	0.236	1	
7-MeC23	Acetone.developed - Methoprene.developed	0.694	3.212	0.002	0.037	*
7-MeC23	Acetone.developed - Precocene.developed	0.124	0.664	0.507	1	
7-MeC23	Methoprene.developed - Queen.developed	-0.684	-2.619	0.016	0.227	
7-MeC25	Acetone.undeveloped - Methoprene.undeveloped	0.73	4.471	0.00001	0.001	* *
7-MeC25	Acetone.undeveloped - Acetone.developed	0.381	3.003	0.003	0.061	
7-MeC25	Acetone.undeveloped - Precocene.undeveloped	-0.013	-0.108	0.914	1	
7-MeC25	Acetone.developed - Methoprene.developed	0.538	4.232	0.00004	0.002	* *

7-MeC25	Acetone.developed - Precocene.developed	-0.008	-0.073	0.942	1	
7-MeC25	Methoprene.developed - Queen.developed	-0.033	-0.213	0.833	1	
7-MeC27	Acetone.undeveloped - Methoprene.undeveloped	-0.104	-1.3	0.196	1	
7-MeC27	Acetone.undeveloped - Acetone.developed	-0.121	-1.934	0.055	0.694	
7-MeC27	Acetone.undeveloped - Precocene.undeveloped	0.002	0.025	0.98	1	
7-MeC27	Acetone.developed - Methoprene.developed	-0.118	-1.89	0.061	0.738	
7-MeC27	Acetone.developed - Precocene.developed	-0.179	-3.309	0.001	0.026	*
7-MeC27	Methoprene.developed - Queen.developed	0.31	2.307	0.039	0.529	
7-MeC29	Acetone.undeveloped - Methoprene.undeveloped	-0.827	-3.967	0.0001	0.004	* *
7-MeC29	Acetone.undeveloped - Acetone.developed	-0.458	-2.815	0.005	0.095	
7-MeC29	Acetone.undeveloped - Precocene.undeveloped	0.125	0.79	0.431	1	
7-MeC29	Acetone.developed - Methoprene.developed	-0.763	-4.735	0.00001	0.001	* *
7-MeC29	Acetone.developed - Precocene.developed	-0.007	-0.05	0.96	1	
7-MeC29	Methoprene.developed - Queen.developed	0.574	3.18	0.003	0.064	
Mix of 8+9+10+11+12-MeC24	Acetone.undeveloped - Methoprene.undeveloped	0.893	4.498	0.00001	0.001	* *
Mix of 8+9+10+11+12-MeC24	Acetone.undeveloped - Acetone.developed	0.52	3.366	0.001	0.022	*
Mix of 8+9+10+11+12-MeC24	Acetone.undeveloped - Precocene.undeveloped	-0.009	-0.059	0.953	1	
Mix of 8+9+10+11+12-MeC24	Acetone.developed - Methoprene.developed	0.648	4.199	0.00004	0.002	* *
Mix of 8+9+10+11+12-MeC24	Acetone.developed - Precocene.developed	0.113	0.844	0.4	1	
Mix of 8+9+10+11+12-MeC24	Methoprene.developed - Queen.developed	-0.093	-0.442	0.664	1	
Mix of 9,19+9,15-diMeC29	Acetone.undeveloped - Methoprene.undeveloped	-0.786	-3.8	0.0002	0.007	* *
Mix of 9,19+9,15-diMeC29	Acetone.undeveloped - Acetone.developed	-0.495	-3.075	0.002	0.051	
Mix of 9,19+9,15-diMeC29	Acetone.undeveloped - Precocene.undeveloped	-0.051	-0.327	0.744	1	
Mix of 9,19+9,15-diMeC29	Acetone.developed - Methoprene.developed	-0.565	-3.509	0.001	0.016	*
Mix of 9,19+9,15-diMeC29	Acetone.developed - Precocene.developed	-0.205	-1.473	0.143	1	
Mix of 9,19+9,15-diMeC29	Methoprene.developed - Queen.developed	0.519	2.54	0.02	0.29	
Mix of 9+11-MeC23	Acetone.undeveloped - Methoprene.undeveloped	0.996	4.089	0.0001	0.003	* *
Mix of 9+11-MeC23	Acetone.undeveloped - Acetone.developed	0.68	3.59	0.0004	0.012	*
Mix of 9+11-MeC23	Acetone.undeveloped - Precocene.undeveloped	-0.138	-0.747	0.456	1	
Mix of 9+11-MeC23	Acetone.developed - Methoprene.developed	0.699	3.685	0.0003	0.01	*
Mix of 9+11-MeC23	Acetone.developed - Precocene.developed	0.156	0.949	0.344	1	
Mix of 9+11-MeC23	Methoprene.developed - Queen.developed	-0.661	-2.79	0.011	0.17	

Mix of 9+11+13-MeC27	Acetone.undeveloped - Methoprene.undeveloped	0.003	0.046	0.963	1	
Mix of 9+11+13-MeC27	Acetone.undeveloped - Acetone.developed	0.104	1.899	0.059	0.729	
Mix of 9+11+13-MeC27	Acetone.undeveloped - Precocene.undeveloped	-0.065	-1.228	0.221	1	
Mix of 9+11+13-MeC27	Acetone.developed - Methoprene.developed	-0.091	-1.672	0.096	1	
Mix of 9+11+13-MeC27	Acetone.developed - Precocene.developed	-0.131	-2.781	0.006	0.102	
Mix of 9+11+13-MeC27	Methoprene.developed - Queen.developed	0.649	4.736	0.0004	0.012	*
n-C22	Acetone.undeveloped - Methoprene.undeveloped	-0.07	-0.457	0.648	1	
n-C22	Acetone.undeveloped - Acetone.developed	0.13	1.084	0.28	1	
n-C22	Acetone.undeveloped - Precocene.undeveloped	-0.084	-0.721	0.472	1	
n-C22	Acetone.developed - Methoprene.developed	0.114	0.959	0.339	1	
n-C22	Acetone.developed - Precocene.developed	0.478	4.611	0.00001	0.001	* * *
n-C22	Methoprene.developed - Queen.developed	0.133	0.622	0.545	1	
n-C23	Acetone.undeveloped - Methoprene.undeveloped	0.021	0.155	0.877	1	
n-C23	Acetone.undeveloped - Acetone.developed	0.19	1.771	0.078	0.901	
n-C23	Acetone.undeveloped - Precocene.undeveloped	-0.151	-1.444	0.151	1	
n-C23	Acetone.developed - Methoprene.developed	0.039	0.367	0.715	1	
n-C23	Acetone.developed - Precocene.developed	0.234	2.518	0.013	0.188	
n-C23	Methoprene.developed - Queen.developed	-0.095	-0.798	0.431	1	
n-C24	Acetone.undeveloped - Methoprene.undeveloped	0.06	0.504	0.615	1	
n-C24	Acetone.undeveloped - Acetone.developed	0.134	1.461	0.146	1	
n-C24	Acetone.undeveloped - Precocene.undeveloped	-0.152	-1.695	0.092	1	
n-C24	Acetone.developed - Methoprene.developed	0.02	0.221	0.825	1	
n-C24	Acetone.developed - Precocene.developed	0.115	1.452	0.149	1	
n-C24	Methoprene.developed - Queen.developed	-0.303	-2.87	0.008	0.124	
n-C25	Acetone.undeveloped - Methoprene.undeveloped	-0.083	-0.773	0.441	1	
n-C25	Acetone.undeveloped - Acetone.developed	0.083	0.997	0.32	1	
n-C25	Acetone.undeveloped - Precocene.undeveloped	-0.041	-0.503	0.616	1	
n-C25	Acetone.developed - Methoprene.developed	-0.148	-1.781	0.077	0.892	
n-C25	Acetone.developed - Precocene.developed	0.072	0.998	0.32	1	
n-C25	Methoprene.developed - Queen.developed	-1.17	-5.714	0.0001	0.003	* *
C25:1	Acetone.undeveloped - Methoprene.undeveloped	0.491	3.446	0.001	0.017	*
C25:1	Acetone.undeveloped - Acetone.developed	0.343	3.094	0.002	0.045	*

C25:1	Acetone.undeveloped - Precocene.undeveloped	-0.183	-1.698	0.091	1	
C25:1	Acetone.developed - Methoprene.developed	0.369	3.34	0.001	0.022	*
C25:1	Acetone.developed - Precocene.developed	0.001	0.008	0.993	1	
C25:1	Methoprene.developed - Queen.developed	-0.534	-3.281	0.006	0.097	
n-C26	Acetone.undeveloped - Methoprene.undeveloped	-0.231	-2.067	0.04	0.54	
n-C26	Acetone.undeveloped - Acetone.developed	-0.026	-0.295	0.769	1	
n-C26	Acetone.undeveloped - Precocene.undeveloped	-0.036	-0.424	0.672	1	
n-C26	Acetone.developed - Methoprene.developed	-0.27	-3.111	0.002	0.046	*
n-C26	Acetone.developed - Precocene.developed	0.008	0.102	0.919	1	
n-C26	Methoprene.developed - Queen.developed	-1.18	-7.097	0.00001	0.001	* *
n-C27	Acetone.undeveloped - Methoprene.undeveloped	-0.368	-2.832	0.005	0.093	
n-C27	Acetone.undeveloped - Acetone.developed	-0.088	-0.868	0.387	1	
n-C27	Acetone.undeveloped - Precocene.undeveloped	0.018	0.186	0.852	1	
n-C27	Acetone.developed - Methoprene.developed	-0.386	-3.828	0.0002	0.006	* *
n-C27	Acetone.developed - Precocene.developed	0.008	0.097	0.923	1	
n-C27	Methoprene.developed - Queen.developed	-1.317	-6.188	0.00004	0.002	* *
C27:1	Acetone.undeveloped - Methoprene.undeveloped	0.071	0.923	0.357	1	
C27:1	Acetone.undeveloped - Acetone.developed	-0.026	-0.435	0.664	1	
C27:1	Acetone.undeveloped - Precocene.undeveloped	-0.087	-1.497	0.135	1	
C27:1	Acetone.developed - Methoprene.developed	0.114	1.91	0.057	0.706	
C27:1	Acetone.developed - Precocene.developed	-0.151	-2.932	0.004	0.068	
C27:1	Methoprene.developed - Queen.developed	-0.035	-0.267	0.794	1	
n-C28	Acetone.undeveloped - Methoprene.undeveloped	-0.416	-2.828	0.005	0.093	
n-C28	Acetone.undeveloped - Acetone.developed	-0.162	-1.417	0.159	1	
n-C28	Acetone.undeveloped - Precocene.undeveloped	0.029	0.262	0.794	1	
n-C28	Acetone.developed - Methoprene.developed	-0.439	-3.841	0.0002	0.007	* *
n-C28	Acetone.developed - Precocene.developed	-0.009	-0.091	0.928	1	
n-C28	Methoprene.developed - Queen.developed	-0.688	-5.182	0.00002	0.001	* *
n-C29	Acetone.undeveloped - Methoprene.undeveloped	-0.748	-4.134	0.0001	0.003	* *
n-C29	Acetone.undeveloped - Acetone.developed	-0.291	-2.065	0.041	0.54	
n-C29	Acetone.undeveloped - Precocene.undeveloped	0.119	0.869	0.386	1	
n-C29	Acetone.developed - Methoprene.developed	-0.657	-4.675	0.00001	0.001	* * *

n-C29	Acetone.developed - Precocene.developed	-0.047	-0.387	0.699	1	
n-C29	Methoprene.developed - Queen.developed	-1.121	-5.366	0.0001	0.004	* *
C29:1	Acetone.undeveloped - Methoprene.undeveloped	-0.147	-1.429	0.155	1	
C29:1	Acetone.undeveloped - Acetone.developed	-0.292	-3.647	0.0004	0.011	*
C29:1	Acetone.undeveloped - Precocene.undeveloped	0.026	0.336	0.737	1	
C29:1	Acetone.developed - Methoprene.developed	0.001	0.009	0.993	1	
C29:1	Acetone.developed - Precocene.developed	0.018	0.254	0.8	1	
C29:1	Methoprene.developed - Queen.developed	-0.301	-1.797	0.097	1	
n-C30	Acetone.undeveloped - Methoprene.undeveloped	-0.298	-2.952	0.004	0.068	
n-C30	Acetone.undeveloped - Acetone.developed	-0.085	-1.083	0.28	1	
n-C30	Acetone.undeveloped - Precocene.undeveloped	-0.07	-0.912	0.363	1	
n-C30	Acetone.developed - Methoprene.developed	-0.259	-3.303	0.001	0.026	*
n-C30	Acetone.developed - Precocene.developed	-0.178	-2.62	0.01	0.148	
n-C30	Methoprene.developed - Queen.developed	0.337	2.137	0.054	0.681	
n-C31	Acetone.undeveloped - Methoprene.undeveloped	-0.649	-3.031	0.003	0.057	
n-C31	Acetone.undeveloped - Acetone.developed	-0.441	-2.644	0.009	0.14	
n-C31	Acetone.undeveloped - Precocene.undeveloped	0.233	1.43	0.155	1	
n-C31	Acetone.developed - Methoprene.developed	-0.319	-1.921	0.057	0.705	
n-C31	Acetone.developed - Precocene.developed	0.041	0.282	0.778	1	
n-C31	Methoprene.developed - Queen.developed	-1.164	-3.451	0.005	0.086	
C31:1	Acetone.undeveloped - Methoprene.undeveloped	-0.321	-1.72	0.087	0.992	
C31:1	Acetone.undeveloped - Acetone.developed	-0.588	-4.051	0.0001	0.003	* *
C31:1	Acetone.undeveloped - Precocene.undeveloped	0.214	1.514	0.132	1	
C31:1	Acetone.developed - Methoprene.developed	0.021	0.145	0.885	1	
C31:1	Acetone.developed - Precocene.developed	0.21	1.675	0.096	1	
C31:1	Methoprene.developed - Queen.developed	-0.424	-2.076	0.056	0.703	

Table S3. Within groups reproductive status contrasts of Aitchison transformed CHC based on linear mixed models with p values fdr corrected according to Benjamini and Yekutieli.

Compound	Contrast	Coefficients	t.ratio	p.value	padj	Significance level
n-C21	Methoprene.developed - Methoprene.undeveloped	-0.15	-1.23	0.5256	1	
n-C21	Precocene.developed - Precocene.undeveloped	-0.53	-4.71	0.0000	0.002	* *
n-C21	Acetone.developed - Acetone.undeveloped	-0.18	-1.47	0.3660	1	
n-C22	Methoprene.developed - Methoprene.undeveloped	-0.31	-2.68	0.0222	0.366	
n-C22	Precocene.developed - Precocene.undeveloped	-0.44	-3.93	0.0003	0.012	*
n-C22	Acetone.developed - Acetone.undeveloped	-0.13	-1.08	0.6239	1	
n-C23	Methoprene.developed - Methoprene.undeveloped	-0.21	-1.99	0.1341	1	
n-C23	Precocene.developed - Precocene.undeveloped	-0.45	-4.54	0.0000	0.002	* *
n-C23	Acetone.developed - Acetone.undeveloped	-0.19	-1.78	0.2107	1	
Mix of 9+11-MeC23	Methoprene.developed - Methoprene.undeveloped	-0.38	-2.06	0.1123	1	
Mix of 9+11-MeC23	Precocene.developed - Precocene.undeveloped	-0.86	-4.85	0.0000	0.002	* *
Mix of 9+11-MeC23	Acetone.developed - Acetone.undeveloped	-0.68	-3.59	0.0010	0.035	*
7-MeC23	Methoprene.developed - Methoprene.undeveloped	-0.31	-1.47	0.3668	1	
7-MeC23	Precocene.developed - Precocene.undeveloped	-0.98	-4.88	0.0000	0.002	* *
7-MeC23	Acetone.developed - Acetone.undeveloped	-0.74	-3.43	0.0018	0.057	
5-MeC23	Methoprene.developed - Methoprene.undeveloped	-0.22	-1.29	0.4825	1	
5-MeC23	Precocene.developed - Precocene.undeveloped	-0.75	-4.53	0.0000	0.002	* *
5-MeC23	Acetone.developed - Acetone.undeveloped	-0.48	-2.71	0.0199	0.345	
3-MeC23	Methoprene.developed - Methoprene.undeveloped	-0.31	-2.47	0.0402	0.605	
3-MeC23	Precocene.developed - Precocene.undeveloped	-0.56	-4.76	0.0000	0.002	* *
3-MeC23	Acetone.developed - Acetone.undeveloped	-0.40	-3.18	0.0044	0.101	
5_9-diMeC23	Methoprene.developed - Methoprene.undeveloped	-0.17	-1.06	0.6434	1	
5_9-diMeC23	Precocene.developed - Precocene.undeveloped	-0.53	-3.56	0.0011	0.037	*
5_9-diMeC23	Acetone.developed - Acetone.undeveloped	-0.43	-2.69	0.0215	0.361	
n-C24	Methoprene.developed - Methoprene.undeveloped	-0.10	-1.06	0.6406	1	
n-C24	Precocene.developed - Precocene.undeveloped	-0.32	-3.75	0.0005	0.022	*
n-C24	Acetone.developed - Acetone.undeveloped	-0.13	-1.46	0.3713	1	
Mix of 3,9+3,7-diMeC23	Methoprene.developed - Methoprene.undeveloped	-0.23	-1.79	0.2041	1	

Mix of 3,9+3,7-diMeC23	Precocene.developed - Precocene.undeveloped	-0.53	-4.22	0.0001	0.005	* *
Mix of 3,9+3,7-diMeC23	Acetone.developed - Acetone.undeveloped	-0.41	-3.06	0.0066	0.138	
Mix of 8+9+10+11+12-MeC24	Methoprene.developed - Methoprene.undeveloped	-0.27	-1.82	0.1933	1	
Mix of 8+9+10+11+12-MeC24	Precocene.developed - Precocene.undeveloped	-0.60	-4.19	0.0001	0.005	* *
Mix of 8+9+10+11+12-MeC24	Acetone.developed - Acetone.undeveloped	-0.52	-3.37	0.0023	0.062	
6-MeC24	Methoprene.developed - Methoprene.undeveloped	-0.10	-0.61	0.9032	1	
6-MeC24	Precocene.developed - Precocene.undeveloped	-0.63	-4.21	0.0001	0.005	* *
6-MeC24	Acetone.developed - Acetone.undeveloped	-0.45	-2.79	0.0158	0.284	
4-MeC24	Methoprene.developed - Methoprene.undeveloped	-0.19	-1.65	0.2676	1	
4-MeC24	Precocene.developed - Precocene.undeveloped	-0.35	-3.19	0.0042	0.101	
4-MeC24	Acetone.developed - Acetone.undeveloped	-0.30	-2.60	0.0277	0.432	
C25:1	Methoprene.developed - Methoprene.undeveloped	-0.22	-1.42	0.4002	1	
C25:1	Precocene.developed - Precocene.undeveloped	-0.65	-4.37	0.0000	0.004	* *
C25:1	Acetone.developed - Acetone.undeveloped	-0.35	-2.15	0.0915	1	
C25:1.y	Methoprene.developed - Methoprene.undeveloped	-0.20	-1.19	0.5530	1	
C25:1.y	Precocene.developed - Precocene.undeveloped	-0.63	-3.96	0.0002	0.011	*
C25:1.y	Acetone.developed - Acetone.undeveloped	-0.39	-2.28	0.0667	0.856	
4,8-diMeC24	Methoprene.developed - Methoprene.undeveloped	-0.26	-1.74	0.2269	1	
4,8-diMeC24	Precocene.developed - Precocene.undeveloped	-0.48	-3.41	0.0019	0.057	
4,8-diMeC24	Acetone.developed - Acetone.undeveloped	-0.42	-2.79	0.0155	0.283	
n-C25	Methoprene.developed - Methoprene.undeveloped	-0.02	-0.22	0.9949	1	
n-C25	Precocene.developed - Precocene.undeveloped	0.00	-0.02	1.0000	1	
n-C25	Acetone.developed - Acetone.undeveloped	-0.08	-1.00	0.6835	1	
Mix of 11+13+15-MeC25	Methoprene.developed - Methoprene.undeveloped	-0.24	-2.67	0.0226	0.368	
Mix of 11+13+15-MeC25	Precocene.developed - Precocene.undeveloped	-0.21	-2.45	0.0427	0.625	
Mix of 11+13+15-MeC25	Acetone.developed - Acetone.undeveloped	-0.39	-4.12	0.0001	0.007	* *
7-MeC25	Methoprene.developed - Methoprene.undeveloped	-0.19	-1.52	0.3362	1	
7-MeC25	Precocene.developed - Precocene.undeveloped	-0.36	-3.06	0.0067	0.138	
7-MeC25	Acetone.developed - Acetone.undeveloped	-0.38	-3.01	0.0079	0.158	
5-MeC25	Methoprene.developed - Methoprene.undeveloped	-0.15	-1.59	0.2979	1	
5-MeC25	Precocene.developed - Precocene.undeveloped	-0.18	-1.97	0.1401	1	
5-MeC25	Acetone.developed - Acetone.undeveloped	-0.30	-3.11	0.0056	0.125	

3-MeC25	Methoprene.developed - Methoprene.undeveloped	-0.10	-2.06	0.1128	1
3-MeC25	Precocene.developed - Precocene.undeveloped	-0.04	-0.78	0.8219	1
3-MeC25	Acetone.developed - Acetone.undeveloped	-0.12	-2.28	0.0666	0.856
5,13-dimeC25	Methoprene.developed - Methoprene.undeveloped	-0.23	-2.36	0.0542	0.751
5,13-dimeC25	Precocene.developed - Precocene.undeveloped	-0.13	-1.34	0.4486	1
5,13-dimeC25	Acetone.developed - Acetone.undeveloped	-0.27	-2.70	0.0206	0.35
n-C26	Methoprene.developed - Methoprene.undeveloped	0.06	0.75	0.8339	1
n-C26	Precocene.developed - Precocene.undeveloped	0.11	1.38	0.4226	1
n-C26	Acetone.developed - Acetone.undeveloped	0.03	0.29	0.9875	1
Mix of 3,9+3,11-diMeC25	Methoprene.developed - Methoprene.undeveloped	-0.20	-2.16	0.0889	1
Mix of 3,9+3,11-diMeC25	Precocene.developed - Precocene.undeveloped	-0.13	-1.45	0.3817	1
Mix of 3,9+3,11-diMeC25	Acetone.developed - Acetone.undeveloped	-0.27	-2.84	0.0135	0.256
12-MeC26	Methoprene.developed - Methoprene.undeveloped	-0.12	-1.94	0.1500	1
12-MeC26	Precocene.developed - Precocene.undeveloped	-0.08	-1.38	0.4260	1
12-MeC26	Acetone.developed - Acetone.undeveloped	-0.18	-2.87	0.0122	0.234
6-Mec26	Methoprene.developed - Methoprene.undeveloped	-0.03	-0.36	0.9773	1
6-Mec26	Precocene.developed - Precocene.undeveloped	-0.10	-1.18	0.5574	1
6-Mec26	Acetone.developed - Acetone.undeveloped	-0.13	-1.40	0.4098	1
4-MeC26	Methoprene.developed - Methoprene.undeveloped	-0.01	-0.13	0.9990	1
4-MeC26	Precocene.developed - Precocene.undeveloped	-0.06	-1.26	0.5026	1
4-MeC26	Acetone.developed - Acetone.undeveloped	0.01	0.29	0.9881	1
C27:1	Methoprene.developed - Methoprene.undeveloped	-0.02	-0.23	0.9942	1
C27:1	Precocene.developed - Precocene.undeveloped	-0.09	-1.15	0.5761	1
C27:1	Acetone.developed - Acetone.undeveloped	0.02	0.26	0.9913	1
C27:1.y	Methoprene.developed - Methoprene.undeveloped	-0.12	-1.05	0.6473	1
C27:1.y	Precocene.developed - Precocene.undeveloped	-0.16	-1.52	0.3407	1
C27:1.y	Acetone.developed - Acetone.undeveloped	-0.01	-0.11	0.9993	1
Mix of4,8+4,6-diMeC26	Methoprene.developed - Methoprene.undeveloped	-0.17	-2.11	0.1016	1
Mix of4,8+4,6-diMeC26	Precocene.developed - Precocene.undeveloped	-0.13	-1.68	0.2553	1
Mix of4,8+4,6-diMeC26	Acetone.developed - Acetone.undeveloped	-0.16	-1.95	0.1458	1
n-C27	Methoprene.developed - Methoprene.undeveloped	0.11	1.07	0.6368	1
n-C27	Precocene.developed - Precocene.undeveloped	0.31	3.26	0.0033	0.086

n-C27	Acetone.developed - Acetone.undeveloped	0.09	0.87	0.7678	1
Mix of 9+11+13-MeC27	Methoprene.developed - Methoprene.undeveloped	-0.01	-0.18	0.9973	1
Mix of 9+11+13-MeC27	Precocene.developed - Precocene.undeveloped	0.07	1.37	0.4264	1
Mix of 9+11+13-MeC27	Acetone.developed - Acetone.undeveloped	-0.10	-1.90	0.1628	1
7-MeC27	Methoprene.developed - Methoprene.undeveloped	0.13	2.19	0.0835	1
7-MeC27	Precocene.developed - Precocene.undeveloped	0.19	3.18	0.0044	0.101
7-MeC27	Acetone.developed - Acetone.undeveloped	0.12	1.93	0.1511	1
5-MeC27	Methoprene.developed - Methoprene.undeveloped	0.04	0.53	0.9344	1
5-MeC27	Precocene.developed - Precocene.undeveloped	-0.01	-0.20	0.9961	1
5-MeC27	Acetone.developed - Acetone.undeveloped	-0.04	-0.54	0.9323	1
11,15-diMeC27	Methoprene.developed - Methoprene.undeveloped	-0.01	-0.09	0.9996	1
11,15-diMeC27	Precocene.developed - Precocene.undeveloped	-0.12	-1.70	0.2445	1
11,15-diMeC27	Acetone.developed - Acetone.undeveloped	-0.07	-0.88	0.7579	1
3-MeC27	Methoprene.developed - Methoprene.undeveloped	0.14	1.40	0.4123	1
3-MeC27	Precocene.developed - Precocene.undeveloped	0.32	3.40	0.0020	0.058
3-MeC27	Acetone.developed - Acetone.undeveloped	0.23	2.24	0.0742	0.94
5,13-diMeC27	Methoprene.developed - Methoprene.undeveloped	-0.02	-0.33	0.9822	1
5,13-diMeC27	Precocene.developed - Precocene.undeveloped	0.05	0.85	0.7807	1
5,13-diMeC27	Acetone.developed - Acetone.undeveloped	-0.04	-0.64	0.8901	1
n-C28	Methoprene.developed - Methoprene.undeveloped	0.19	1.66	0.2643	1
n-C28	Precocene.developed - Precocene.undeveloped	0.34	3.20	0.0041	0.101
n-C28	Acetone.developed - Acetone.undeveloped	0.16	1.42	0.3986	1
Mix of 3,11+3,13- diMeC27	Methoprene.developed - Methoprene.undeveloped	-0.05	-0.97	0.7047	1
Mix of 3,11+3,13- diMeC27	Precocene.developed - Precocene.undeveloped	0.10	1.91	0.1584	1
Mix of 3,11+3,13- diMeC27	Acetone.developed - Acetone.undeveloped	-0.10	-1.88	0.1700	1
Mix of 10+12+14+16-MeC28	Methoprene.developed - Methoprene.undeveloped	0.07	0.89	0.7564	1
Mix of 10+12+14+16-MeC28	Precocene.developed - Precocene.undeveloped	0.22	3.02	0.0076	0.154
Mix of 10+12+14+16-MeC28	Acetone.developed - Acetone.undeveloped	-0.04	-0.56	0.9238	1
4-MeC28	Methoprene.developed - Methoprene.undeveloped	0.22	2.20	0.0818	1
4-MeC28	Precocene.developed - Precocene.undeveloped	0.15	1.63	0.2761	1
4-MeC28	Acetone.developed - Acetone.undeveloped	0.26	2.60	0.0279	0.432
C29:1	Methoprene.developed - Methoprene.undeveloped	0.14	1.84	0.1856	1

C29:1	Precocene.developed - Precocene.undeveloped	0.18	2.37	0.0519	0.739	
C29:1	Acetone.developed - Acetone.undeveloped	0.29	3.65	0.0008	0.029	*
Mix of 4,12+4,14-diMeC28	Methoprene.developed - Methoprene.undeveloped	-0.01	-0.15	0.9984	1	
Mix of 4,12+4,14-diMeC28	Precocene.developed - Precocene.undeveloped	0.09	1.38	0.4219	1	
Mix of 4,12+4,14-diMeC28	Acetone.developed - Acetone.undeveloped	0.02	0.27	0.9902	1	
n-C29	Methoprene.developed - Methoprene.undeveloped	0.20	1.45	0.3810	1	
n-C29	Precocene.developed - Precocene.undeveloped	0.59	4.48	0.0000	0.002	* *
n-C29	Acetone.developed - Acetone.undeveloped	0.29	2.07	0.1122	1	
Mix of 11+13+15-MeC29	Methoprene.developed - Methoprene.undeveloped	0.18	1.67	0.2566	1	
Mix of 11+13+15-MeC29	Precocene.developed - Precocene.undeveloped	0.42	4.19	0.0001	0.005	* *
Mix of 11+13+15-MeC29	Acetone.developed - Acetone.undeveloped	0.25	2.31	0.0608	0.82	
7-MeC29	Methoprene.developed - Methoprene.undeveloped	0.39	2.49	0.0380	0.581	
7-MeC29	Precocene.developed - Precocene.undeveloped	0.62	4.11	0.0001	0.007	* *
7-MeC29	Acetone.developed - Acetone.undeveloped	0.46	2.82	0.0142	0.264	
5-MeC29	Methoprene.developed - Methoprene.undeveloped	0.26	1.89	0.1653	1	
5-MeC29	Precocene.developed - Precocene.undeveloped	0.31	2.36	0.0540	0.751	
5-MeC29	Acetone.developed - Acetone.undeveloped	0.31	2.21	0.0796	0.985	
Mix of 9,19+9,15-diMeC29	Methoprene.developed - Methoprene.undeveloped	0.27	1.74	0.2271	1	
Mix of 9,19+9,15-diMeC29	Precocene.developed - Precocene.undeveloped	0.37	2.45	0.0418	0.62	
Mix of 9,19+9,15-diMeC29	Acetone.developed - Acetone.undeveloped	0.49	3.08	0.0063	0.135	
3-MeC29	Methoprene.developed - Methoprene.undeveloped	0.32	1.91	0.1592	1	
3-MeC29	Precocene.developed - Precocene.undeveloped	0.73	4.58	0.0000	0.002	* *
3-MeC29	Acetone.developed - Acetone.undeveloped	0.62	3.54	0.0012	0.039	*
5,9-diMeC29	Methoprene.developed - Methoprene.undeveloped	0.10	0.95	0.7124	1	
5,9-diMeC29	Precocene.developed - Precocene.undeveloped	0.22	2.29	0.0653	0.856	
5,9-diMeC29	Acetone.developed - Acetone.undeveloped	0.12	1.19	0.5536	1	
n-C30	Methoprene.developed - Methoprene.undeveloped	0.05	0.60	0.9098	1	
n-C30	Precocene.developed - Precocene.undeveloped	0.21	2.92	0.0105	0.207	
n-C30	Acetone.developed - Acetone.undeveloped	0.09	1.08	0.6246	1	
Mix of10+11+12+13+14-MeC30	Methoprene.developed - Methoprene.undeveloped	0.31	2.44	0.0437	0.63	
Mix of10+11+12+13+14-MeC30	Precocene.developed - Precocene.undeveloped	0.49	3.99	0.0002	0.01	*
Mix of10+11+12+13+14-MeC30	Acetone.developed - Acetone.undeveloped	0.45	3.40	0.0020	0.058	

C31:1	Methoprene.developed - Methoprene.undeveloped	0.25	1.73	0.2303	1	
C31:1	Precocene.developed - Precocene.undeveloped	0.44	3.23	0.0037	0.094	
C31:1	Acetone.developed - Acetone.undeveloped	0.59	4.05	0.0002	0.008	* *
n-C31	Methoprene.developed - Methoprene.undeveloped	0.11	0.68	0.8727	1	
n-C31	Precocene.developed - Precocene.undeveloped	0.61	3.89	0.0003	0.014	*
n-C31	Acetone.developed - Acetone.undeveloped	0.44	2.64	0.0244	0.39	
Mix of 11+13+15-MeC31	Methoprene.developed - Methoprene.undeveloped	0.31	1.72	0.2340	1	
Mix of 11+13+15-MeC31	Precocene.developed - Precocene.undeveloped	0.78	4.61	0.0000	0.002	* *
Mix of 11+13+15-MeC31	Acetone.developed - Acetone.undeveloped	0.65	3.57	0.0011	0.037	*
Mix of 11,17+13,17+15,19-diMeC31	Methoprene.developed - Methoprene.undeveloped	0.54	2.30	0.0626	0.833	
Mix of 11,17+13,17+15,19-diMeC31	Precocene.developed - Precocene.undeveloped	0.85	3.88	0.0003	0.014	*
Mix of 11,17+13,17+15,19-diMeC31	Acetone.developed - Acetone.undeveloped	0.89	3.72	0.0006	0.024	*
3-MeC31	Methoprene.developed - Methoprene.undeveloped	0.27	1.60	0.2951	1	
3-MeC31	Precocene.developed - Precocene.undeveloped	0.69	4.20	0.0001	0.005	* *
3-MeC31	Acetone.developed - Acetone.undeveloped	0.60	3.42	0.0019	0.057	
5,13-diMeC31	Methoprene.developed - Methoprene.undeveloped	0.03	0.31	0.9858	1	
5,13-diMeC31	Precocene.developed - Precocene.undeveloped	0.31	3.14	0.0051	0.115	
5,13-diMeC31	Acetone.developed - Acetone.undeveloped	0.23	2.21	0.0783	0.98	
13+15-MeC33	Methoprene.developed - Methoprene.undeveloped	0.28	1.97	0.1394	1	
13+15-MeC33	Precocene.developed - Precocene.undeveloped	0.62	4.47	0.0000	0.002	* *
13+15-MeC33	Acetone.developed - Acetone.undeveloped	0.41	2.77	0.0165	0.291	
11,21-diMeC33	Methoprene.developed - Methoprene.undeveloped	0.56	2.33	0.0588	0.803	
11,21-diMeC33	Precocene.developed - Precocene.undeveloped	0.84	3.68	0.0007	0.027	*
11,21-diMeC33	Acetone.developed - Acetone.undeveloped	0.83	3.36	0.0024	0.063	

Table S4. Raw data survival analysis. Methoprene treatment concentrations, M1=5 µg/µl, M2=10 µg/µl, M3=20 µg/µl, and Precocene treatment concentrations, P1=2 µg/µl, P2=6 µg/µl, P3=15 µg/µl.

**Group**	**Alive**	**Dead**	**Days**
Untreated	10	0	0
Aceton	10	0	0
M1	10	0	0
M2	10	0	0
M3	10	0	0
P1	8	0	0
P2	9	0	0
P3	10	0	0
Untreated	10	0	3
Aceton	9	1	3
M1	8	2	3
M2	9	1	3
M3	6	4	3
P1	7	1	3
P2	7	2	3
P3	9	1	3
Untreated	9	1	6
Aceton	8	2	6
M1	8	2	6
M2	6	4	6
M3	6	4	6
P1	5	3	6
P2	6	3	6
P3	9	1	6

Table S5. Pairwise hazard ratio contrasts derived from Cox proportional hazards regression model. P values were Tukey fdr corrected.

Contrast	Hazard ratio	SE	z ratio	p value
aceton - M1	0.894	0.894	-0.112	1.000
aceton - M2	0.449	0.389	-0.923	0.984
aceton - M3	0.37	0.321	-1.147	0.946
aceton - not_treated	2.048	2.508	0.585	0.999
aceton - P1	0.476	0.434	-0.814	0.992
aceton - P2	0.51	0.466	-0.737	0.996
aceton - P3	1.942	2.378	0.542	0.999
M1 - M2	0.503	0.436	-0.794	0.993
M1 - M3	0.414	0.359	-1.018	0.972
M1 - not_treated	2.291	2.806	0.677	0.998
M1 - P1	0.532	0.486	-0.691	0.997
M1 - P2	0.571	0.521	-0.615	0.999
M1 - P3	2.172	2.661	0.633	0.998
M2 - M3	0.823	0.582	-0.275	1.000
M2 - not_treated	4.556	5.095	1.356	0.877
M2 - P1	1.058	0.808	0.074	1.000
M2 - P2	1.135	0.867	0.166	1.000
M2 - P3	4.32	4.831	1.308	0.896
M3 - not_treated	5.537	6.193	1.53	0.791
M3 - P1	1.286	0.982	0.329	1.000
M3 - P2	1.379	1.053	0.421	1.000
M3 - P3	5.25	5.872	1.482	0.817
not_treated - P1	0.232	0.268	-1.264	0.912
not_treated - P2	0.249	0.288	-1.204	0.931
not_treated - P3	0.948	1.341	-0.038	1.000
P1 - P2	1.073	0.876	0.086	1.000
P1 - P3	4.083	4.716	1.218	0.927
P2 - P3	3.807	4.397	1.158	0.944
